# The Evolution and Biological Activity of Metazoan Mixed Lineage Kinase Domain-Like Protein (MLKL)

**DOI:** 10.3390/ijms251910626

**Published:** 2024-10-02

**Authors:** Qingyue Wang, Zihao Yuan, Hang Xu, Yuan Chen, Li Sun

**Affiliations:** 1 CAS and Shandong Province Key Laboratory of Experimental Marine Biology, Institute of Oceanology, Center for Ocean Mega-Science, Chinese Academy of Sciences, Qingdao 266404, China; 2 Laboratory for Marine Biology and Biotechnology, Qingdao Marine Science and Technology Center, Qingdao 266237, China; 3 College of Marine Sciences, University of Chinese Academy of Sciences, Qingdao 266404, China

**Keywords:** MLKL, necroptosis, evolution

## Abstract

In mammals, mixed lineage kinase domain-like protein (MLKL) is the executor of necroptosis. MLKL comprises an N-terminal domain (NTD), which alone suffices to trigger necroptosis by forming pores in the plasma membrane, and a C-terminal domain that inhibits the NTD activity. Evolutionarily, MLKL is poorly conserved in animals and not found in Protostomia. Although MLKL orthologs exist in invertebrate Deuterostomia, the biological activity of invertebrate MLKL is unknown. Herein, we examined 34 metazoan phyla and detected MLKL not only in Deuterostomia but also in Protostomia (Rotifera). The Rotifera MLKL exhibited low identities with non-Rotifera MLKL but shared relatively high identities with non-metazoan MLKL. In invertebrates, MLKL formed two phylogenetic clades, one of which was represented by Rotifera. In vertebrates, MLKL expression was tissue-specific and generally rich in immune organs. When expressed in human cells, the MLKL-NTD of Rotifera, Echinodermata, Urochordata, and Cephalochordata induced strong necroptosis. The necroptotic activity of Rotifera MLKL depended on a number of conserved residues. Together these findings provided new insights into the evolution of MLKL in Metazoa and revealed the biological activity of invertebrate MLKL.

## 1. Introduction

Necroptosis is a form of programmed cell death that plays an important role in innate immunity in mammals [1,2,3,4,5]. Necroptosis is triggered by activation of death domain-containing receptors, e.g., tumor necrosis receptor 1 (TNFR1), Toll-like receptors (TLR), and the Z-DNA-recognizing molecule Z-DNA Binding Protein 1 (ZBP1) (also called DNA Activator of Interferon, DAI) [6,7,8,9]. Via adaptors such as TNFR1-associated death domain protein (TRADD), the activated death receptors recruit receptor-interacting serine/threonine protein kinase (RIPK) 1. RIPK1 activation leads to either proinflammatory response mediated by the NF-κB and MAPK (mitogen-activated protein kinase) pathways or programmed cell death. In the latter case, RIPK1 activates caspase 8 to induce apoptosis, or, when caspase 8 is not available, it activates RIPK3 by phosphorylation. RIPK3 then recruits mixed lineage kinase domain-like protein (MLKL), forming the complex called necrosome, in which MLKL is activated by RIPK3 phosphorylation. The activated MLKL mediates necroptosis by forming large pores in the plasma membrane, resulting in membrane rupture and cell death. In humans and mice, necroptosis triggers the release of inflammatory cytokines and DAMPs (Danger-Associated Molecular Patterns), which recruit immune cells and induce immune responses to facilitate pathogen killing or tissue repairing [10].

MLKL contains an N-terminal domain (NTD) that forms a four-helix bundle (4HB), a C-terminal domain (CTD) of pseudokinase, and a connecting brace featuring a two-helix bundle [11,12]. The mechanism of MLKL that causes cell death is complex, and different MLKL membrane pore-forming mechanisms have been proposed [13]. In general, the NTD is the pore-forming effector and alone can cause membrane rupture. In healthy cells, MLKL exists in the cytoplasm as inactive monomers, in which the membrane-targeting activity of the NTD is auto-inhibited by the CTD. During necroptosis, MLKL is phosphorylated by RIPK3 at the CTD (Thr357/Ser358 in humans and Ser345/Ser347 in mice), which induces a conformational change in the pseudokinase domain, thus revealing the pore-forming 4HB domain [14,15,16,17,18]. The phosphorylated MLKL molecules form oligomers that target the plasma membrane via interaction between the 4HB and the phosphatidylinositol phosphates (PIPs) in the plasma membrane [19,20,21]. In the membrane, MLKL oligomers form destructive cationic channels that span the membrane, leading to cell swelling and eventually necroptotic cell death due to membrane damage [14,15,16,17].

To date, the research on MLKL function is limited mainly to Mammalia represented by humans and mice. Whether functional MLKL exists in other animal phyla is obscure. Evolutionary studies of MLKL are also limited. A research of MLKL distribution in 51 species indicated poor conservation of MLKL in the animal kingdom [22]. For example, in vertebrates, MLKL was absent in zebrafish *Danio rerio* based on the reference genome. In invertebrates, MLKL orthologs were detected in Echinodermata, Hemichordata, and Chordata, but were not found in any phylum belonging to Protostomia [22]. As such, the origin of MLKL is unclear, and the biological function of metazoan MLKL is unknown. In non-Metazoa, MLKL homologs are present in fungi and plants [23,24,25]; however, their relationships with metazoan MLKL are not clear.

In this study, through in-depth data mining, we investigated the distribution of MLKL across Metazoa and explored the expression profile and biological activity of the identified MLKL orthologs. We detected MLKL orthologs in both Protostomia (Rotifera) and Deuterostomia and determined the necroptotic activity of invertebrate MLKL, especially the relatively more primitive MLKL from Rotifera. Our results revealed the fundamental functions of invertebrate MLKL and provided new insights into MLKL evolution in Metazoa.

## 2. Results

### 2.1. Divergent Evolution of MLKL in Invertebrates and Vertebrates

**Figure 1:**
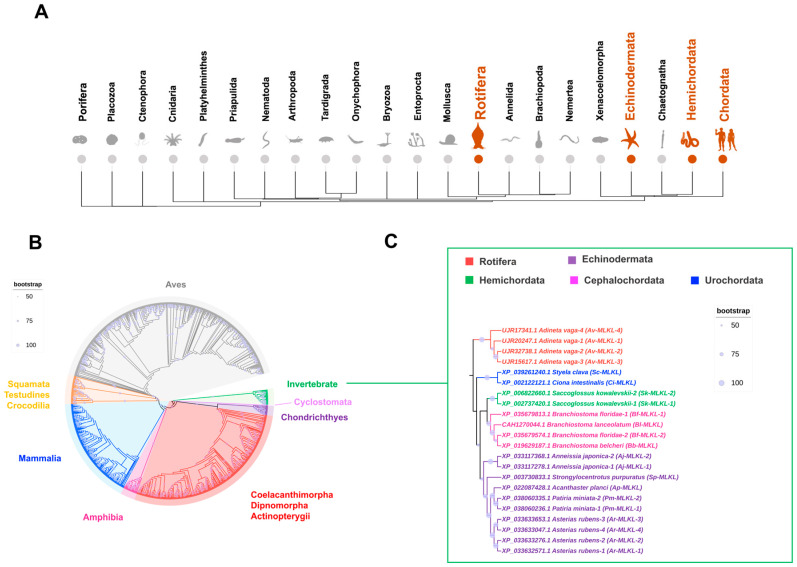
Phylogenetic analysis of MLKL in Metazoa. (**A**) The phylogenetic tree of the major metazoan phyla with MLKL distribution. Orange and grey indicate the presence and absence of MLKL, respectively. (**B**) The phylogeny of invertebrate and vertebrate MLKL. The phylogenetic tree was constructed via maximum likelihood analysis with the JTT + F + R9 substitution model implemented in IQ-TREE 2 v.2.1.2. Invertebrate MLKL was used as an outgroup. The bootstraps are indicated. Different clades are colored differently. (**C**) Phylogenetic analysis of invertebrate MLKL. The phylogenetic tree was constructed via maximum likelihood analysis with the VT + F + I + G4 substitution model implemented in IQ-TREE 2 v.2.1.2. The tree was rooted at midpoint, and the bootstraps are indicated. Organism silhouettes were collected from PhyloPic (www.phylopic.org), accessed on 1 October 2023.

A data-driven approach was used to scan 34 metazoan phyla for potential MLKL. The result showed that Radiata and Ecdysozoa lacked MLKL. However, in Lophotrochozoa, although most phyla lacked MLKL, Rotifera possessed MLKL, all of which were from *Adineta vaga* and were named AvMLKL-1 to 4. No RIPK homolog was identified in Rotifera. In invertebrates, in addition to Rotifera, MLKL was also present in Echinodermata, Hemichordata, and Chordata (Figure 1A). In total, 868 MLKL were identified, of which 22 were from invertebrate species. Invertebrate MLKL shared a mean identity of 25.19% with vertebrate MLKL, based on pairwise comparison, and formed a single phylogenetic clade clearly separated from those of vertebrate MLKL (Figure 1B, Supplemental Table S1). In vertebrates, Cyclostomata was the ancestor to all jawed vertebrates in the MLKL phylogeny. In jawed vertebrates, fish (Actinopterygii, Chondrichthyes, Coelacanthimorpha, and Dipnomorpha) MLKL formed the root group of tetrapod MLKL. In Tetrapoda, Amphibia constituted the basal clade to the Amniota clade. Of the fish MLKL, Coelacanthimorpha, Dipnomorpha, and Actinopterygii clustered into a group distinct from the group of Chondrichthyes (Figure 1B). In invertebrates, MLKL evolved into two general clades, one of which was composed of Rotifera, and the other was composed of all the other invertebrate species (Echinodermata, Hemichordata, Cephalochordata, and Urochordata) (Figure 1C). In the non-Rotifera clade, Hemichordata and Cephalochordata formed a group distinct from the Echinodermata group, and both groups were diverged from Urochordata (Figure 1C). The Rotifera and non-Rotifera MLKL shared a mean identity of 18.77%. In contrast, some rotiferian MLKL exhibited relatively high levels of identity with non-metazoan MLKL. For example, AvMLKL-2 (UJR32738.1) shared 31.1% identity with *Zingiber officinale* (plant) MLKL (XP_042412471.1), and AvMLKL-4 (UJR17341.1) shared 24.5% identity with *Basidiobolus meristosporus* (fungi) MLKL (ORX91397.1). Phylogeny analysis of invertebrate MLKL with non-metazoan MLKL showed that Rotifera MLKL clustered with fungi/plant MLKL (Supplemental Figure S1).

### 2.2. Variation in MLKL Gene Number in Invertebrates and Vertebrates

**Figure 2:**
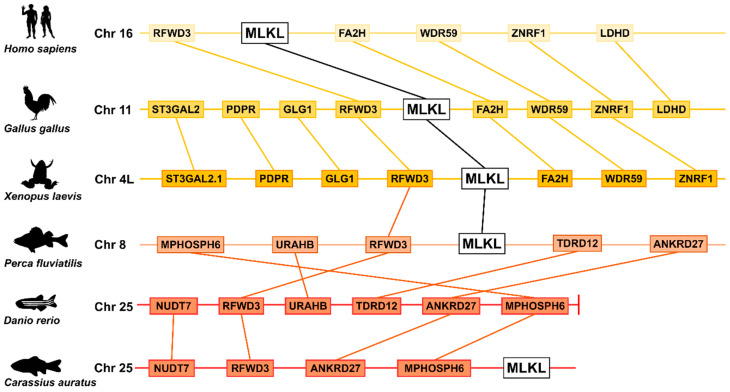
The synteny blocks around vertebrate MLKL. The schematic diagram showing the conserved neighbor genes of MLKL in representative Actinopterygii (*Perca fluviatilis*, *Danio rerio*, and *Carassius auratus*), Mammalia (*Homo sapiens*), Aves (*Gallus gallus*), and Amphibia (*Xenopus laevis*). Organism silhouettes were collected from PhyloPic (www.phylopic.org), accessed on 1 October 2023.

In general, tetrapods had one MLKL gene. In contrast, Rotifera *A. vaga* had four copies of MLKL (Figure 1C, Supplemental Tables S1 and S2). In Actinopterygii, 68.9% (120 out of 174) of the species possessed one MLKL, and 31.1% of the species possessed two to four copies of MLKL, e.g., some Cyprinidae (such as *Puntigrus tetrazona* and *Carassius gibelio*) and *Salmo* species had two or three MLKL, while *Thunnus maccoyii* and the Arctic species of *Dissostichus mawsoni* and *Trematomus bernacchii* had three or four MLKL (Supplemental Tables S1 and S2). MLKL was absent in some fish, mostly non-Percomorphaceae Euteleosteomorpha species, such as those from the orders of Ateleopodiformes, Aulopiformes, Myctophiformes, Lampriformes, Percopsiformes, Zeiformes, and Stylephoriformes. Most of these fish inhabit deep seas, suggesting a link between the deep-sea environment and the loss of MLKL. However, zebrafish (*Danio rerio*), a member of Cyprinidae and a model species used for many biological studies, lacks MLKL. Synteny analysis showed that the neighbor genes of MLKL were conserved in the teleost *C. carpio* and *Perca fluviatilis* and in the tetrapod *Xenopus laevis*, *Gallus gallus*, and *Homo sapiens* (Figure 2). In contrast, in zebrafish, these neighbor genes, such as RFWD3 and ANKRD27, were altered in position and localized at the end of the chromosome. 

### 2.3. Tissue Specific Expression Profiles of Vertebrate MLKL

**Figure 3:**
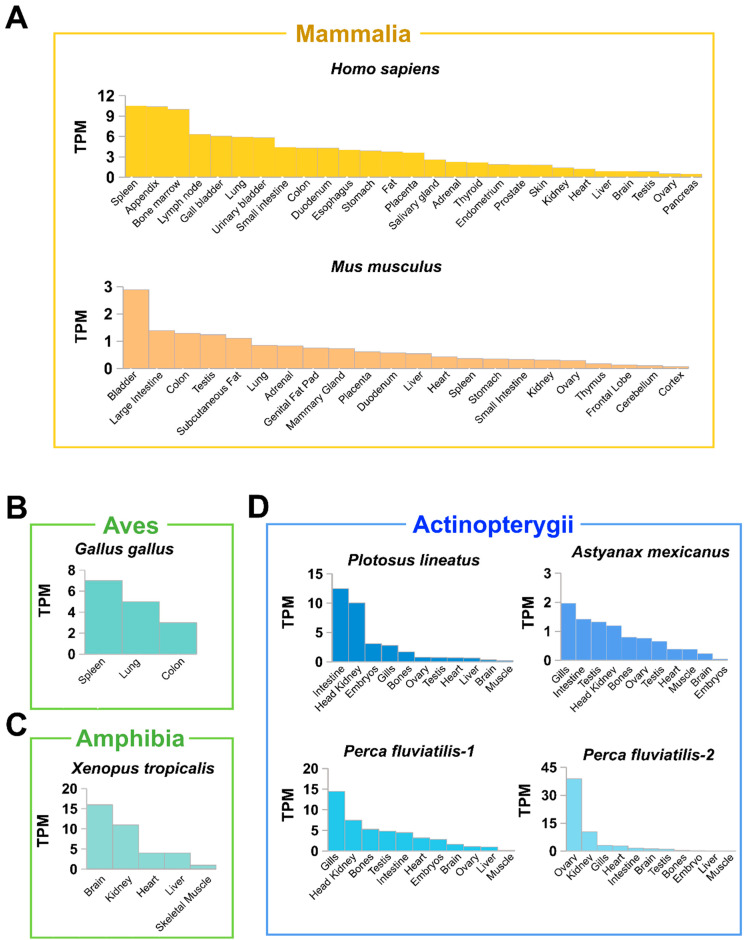
Tissue expression profiles of MLKL in transcript per million (TPM). Expression diagrams depicting the tissue expression profiles of the MLKL in representative (**A**) Mammalia (*Homo sapiens* and *Mus musculus*), (**B**) Aves (*Gallus gallus*), (**C**) Amphibia (*Xenopus tropicalis*), and (**D**) Actinopterygii, i.e., *Pangasianodon hypophthalmus*, *Astyanax mexicanus*, *Perca fluviatilis*-1 (XP_039665593.1), and *Perca fluviatilis*-2 (XP_039665591.1).

To better understand the biological role of MLKL, the tissue-specific expression profiles of MLKL were examined in the major classes of vertebrates, ranging from fish to mammals. In Mammalia, human *Homo sapiens* MLKL (HsMLKL) was abundantly expressed in the spleen, appendix, bone marrow, and lymph nodes, while mouse *Mus musculus* MLKL (MmMLKL) was highly expressed in the bladder (Figure 3A). In Aves, MLKL expression was most abundant in the spleen and lung of chickens (Figure 3B). In Amphibia, MLKL expression was rich in the brain and kidney of frogs (Figure 3C). In fish, *Pangasianodon hypophthalmus* and *Astyanax mexicanus*, which had one MLKL gene, exhibited high levels of MLKL expression in immune organs, such as the intestine, head kidney, and gills. However, in *P. fluviatilis*, which possessed two copies of MLKL, one MLKL was highly expressed in gills, while the other MLKL was highly expressed in the ovary (Figure 3D), suggesting functional compartmentalization of different MLKL genes. 

### 2.4. Necroptosis-Inducing Abilities of Invertebrate MLKL

**Figure 4:**
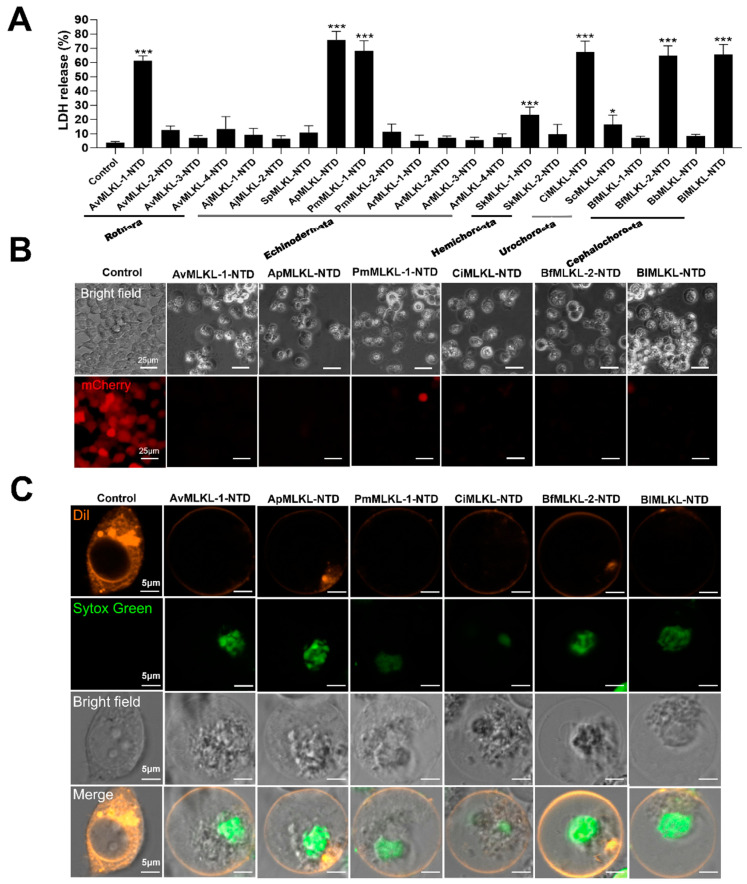
The cell death-inducing ability of invertebrate MLKL. (**A**) HEK293T cells were transfected with the backbone vector (control) or the vectors expressing 22 mCherry-tagged invertebrate MLKL-NTD for 24 h, and LDH release was determined. The abbreviations of the MLKL are shown in Figure 1C. Data are the means ± SD of triplicate experiments. * *p* < 0.05; *** *p* < 0.001. (**B**) Microscopic images of HEK293T cells carrying the control vector or the vector expressing mCherry-tagged MLKL-NTD with necroptotic activity. Scale bar, 25 μm. (**C**) HEK293T cells were transfected with the control vector or the vector expressing Flag-tagged MLKL-NTD with necroptotic activity. The cell membrane and nucleus were stained with Dil and Sytox green, respectively. Scale bar, 5 μm.

To date, no report on the biological activity of invertebrate MLKL has been documented. In this study, we examined the necroptosis-inducing potentials of the 22 invertebrate MLKL identified in Urochordata (*Ciona intestinalis* and *Styela clava*), Cephalochordata (*Branchiostoma floridae*, *Branchiostoma belcheri*, and *Branchiostoma lanceolatum*), Hemichordata (*Saccoglossus kowalevskii*), Echinodermata (*Patiria miniata*, *Acanthaster planci*, *Anneissia japonica*, *Asterias rubens*, and *Strongylocentrotus purpuratus*), and Rotifera (*Adineta vaga*). Since the N-terminal domain (NTD) containing the 4HB of MLKL is the actual executor of necroptosis, the NTD of the 22 MLKL were each expressed in HEK293T cells, and the viability of the cells was subsequently examined. Robust cell death and lactate dehydrogenase (LDH) release (an indicator of necroptosis) were induced by the MLKL-NTD of Rotifera (*A. vaga*), Urochordata (*C. intestinalis*), Cephalochordata (*B. floridae* and *B. lanceolatum*), and Echinodermata (*A. planci* and *P. miniata*) at as early as 12 h post-transfection (Figure 4A; Supplemental Figure S2). The dying cells exhibited typical features of necroptotic cell death, including cell swelling and plasma membrane bubbling (Figure 4B,C). These results indicated that some invertebrate MLKL possessed necroptosis-inducing capacity.

### 2.5. Identification of Key Residues Required for the Necroptotic Activity of AvMLKL-1

**Figure 5:**
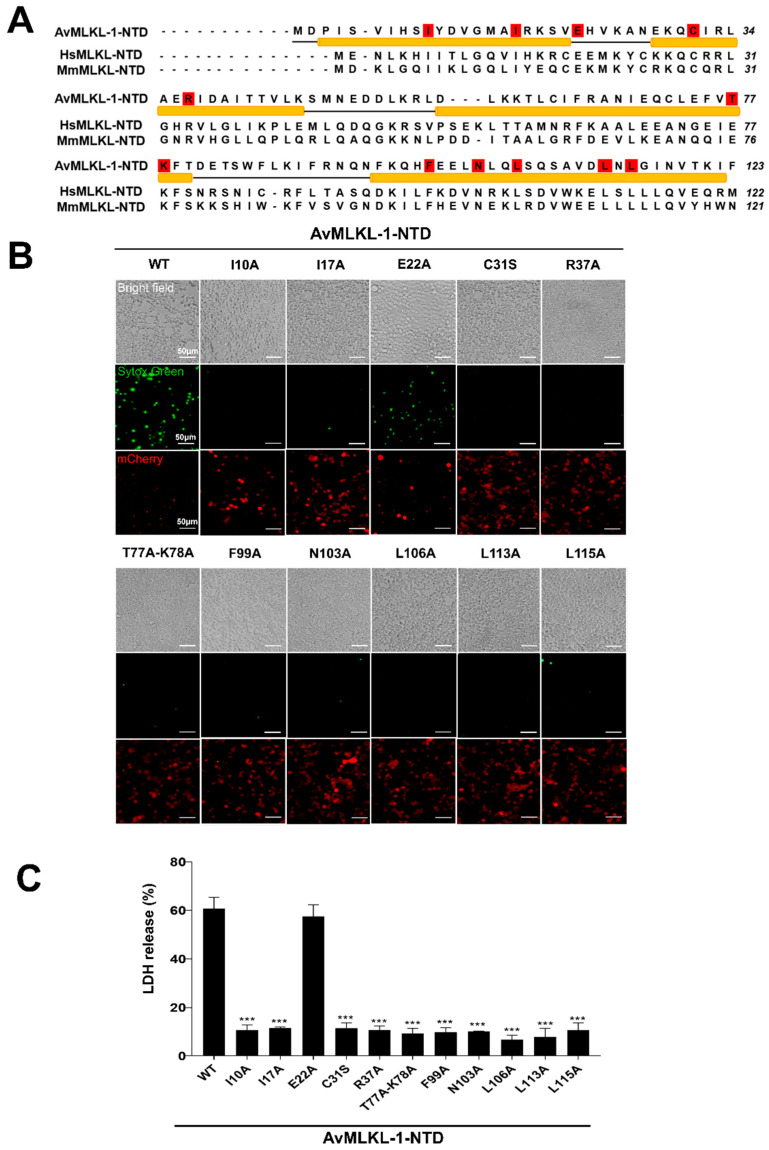
Involvement of the conserved residues in the necroptotic activity of AvMLKL-1. (**A**) Sequence alignment of AvMLKL-1, HsMLKL, and MmMLKL. The alpha helices in AvMLKL-1 are indicated by orange bars. The residues used for mutagenesis are highlighted in red. (**B**) HEK293T cells were transfected with the vector expressing wild type (WT) or mutant AvMLKL-1 NTD (C-terminally tagged with mCherry) for 24 h. The cells were stained with Sytox green and observed with a fluorescence microscope. Scale bar, 50 μm. (**C**) LDH release from the cells of (**B**) was measured. Data are the means ± SD of triplicate experiments. *** *p* < 0.001.

Of the above examined invertebrate species with marked necroptotic activity, Rotifera *A. vaga* is the most primitive. Sequence analysis showed that the four *A. vaga* MLKL had a mean pairwise identity of 32.78%. In humans, the pore-forming activity of MLKL NTD is inhibited by the CTD. As a result, the full-length MLKL is an inactive protein. Similarly, none of the four *A. vaga* MLKL full-length proteins exhibited cytotoxicity (Supplemental Figure S3). Since, as shown above, the NTD of AvMLKL-1 induced robust necroptotic cell death, we searched for the key residues of AvMLKL-1 involved in necroptotic activity. AvMLKL-1 NTD shared 20.9% identity with mouse MLKL-NTD and contained a number of conserved residues in the four α-helices (Figure 5A). Twelve AvMLKL-1 residues, i.e., I10, I17, E22, C31, R37, T77-K78, F99, N103, L106, L113, and L115, corresponded to the murine MLKL residues located in the 4HB structure and were conserved among rotiferian and mammalian MLKL-NTD [26]. These 12 residues were mutated to examine their functional importance (Figure 5A). The results showed that except for the E22A mutant, all the other 10 mutants, i.e., I10A, I17A, C31S, R37A, T77A-K78A (T77A and K78A double mutations), F99A, N103A, L106A, L113A, and L115A, were severely impaired in the ability to induce cell death, despite their high expression levels (Figure 5B). Consistently, LDH release caused by these 10 mutants was significantly reduced (Figure 5C).

## 3. Discussion

In the present work, we performed an in-depth search of MLKL in 34 metazoan phyla. We found that MLKL orthologs existed in both Protostomia and Deuterostomia. In Protostomia, MLKL was discovered only in Bdelloidea Rotifera, and was not detected in any of the other Protostomia, including those with complex and diverse innate immune systems, such as Spiralia Mollusca [27] and Ecdysozoa Arthropoda [28]. This poor conservation of MLKL in the major Protostomia and the more basal groups disfavors MLKL as a fundamentally essential protein in early evolution. Phylogenetic evidence supported a monophyletic Rotifera MLKL clade that was separated from all the other invertebrate Deuterostomia MLKL, including those from Echinodermata, Hemichordata, Cephalochordata, and Urochordata, implying different paths taken by Protostomia and Deuterostomia in MLKL evolution. It is noteworthy that bdelloid Rotifera MLKL shared relatively higher identities with plant/fungi MLKL. Previous studies on bdelloid rotifer genomes revealed that compared with other Lophotrochozoa, Bdelloidea contained an extremely high number of unique gene families that were absent in other metazoan phyla and derived via massive horizontal gene transfer from bacteria, fungi, and plants [29,30]. Given these observations, it is possible that bdelloid rotifers may have acquired the MLKL genes from non-metazoans by horizontal gene transfer. Taken together, these results provide new insights into the evolution and divergence of metazoan MLKL.

Vertebrate MLKL constituted the major portion of the identified MLKL in our study. Actinopterygii is the largest and most successful vertebrate group, making up over half of the existing vertebrate species [31]. In Actinopterygii, we found that some species possessed multiple copies of MLKL, which may be the consequence of the teleost-specific genome duplication and/or an additional whole-genome duplication event [32,33,34]. However, in other species, notably the model organism *D. rerio*, MLKL was absent. Synteny block analysis showed that, unlike that in the MLKL-positive cyprinid and tetrapods, in *D. rerio* the MLKL neighboring genes were located at the chromosome terminus, suggesting the possibility of MLKL loss due to frequently occurring chromosomal recombination in the region adjacent to the telomeres [35]. Although *D. rerio* possesses RIPK3 [22], the absence of MLKL suggests that, as proposed in a previous study [36], *D. rerio* technically lacks necroptosis. In line with the known involvement of MLKL in immunity in mammals [37,38], MLKL expressions in fish and tetrapods were abundant in immune organs, suggesting that MLKL may, like that in mammals, play an immune-related role in non-mammalian vertebrates.

To date, no study on the function of invertebrate MLKL has been documented. In this work, we analyzed the cytotoxic potential of 22 MLKL from 12 invertebrate species. We found that robust necroptotic death of human cells was induced by the MLKL-NTD of Rotifera, Echinodermata, Cephalochordata, and Urochordata. This finding indicated that the pore-forming activity of MLKL is conserved throughout evolution from invertebrate to vertebrate, which is in agreement with a previous study of vertebrate MLKL showing that the membrane permeabilization function of MLKL-NTD is evolutionarily conserved [39]. Previous reports have shown that although mouse MLKL (1-180) cannot induce human cell death [39], cross-species necroptotic activities have been observed in several MLKL. For example, the NTDs of horse and frog MLKL can induce effective necroptosis in mouse cells [39]. The activation mechanisms of MLKL from different species may differ and need to be explored. In our study, we observed apparent necroptosis of human cells induced by some invertebrate MLKL-NTD, suggesting that either these MLKL-NTD did not require any activation modification from the host human cells or the required modification could be provided by the human cells. The exact mechanism needs to be studied in the future. The four-helix bundle of MLKL-NTD is essential for membrane targeting [26,40]. In our study, we found that similar major α-helix structures existed in the NTD of the primitive Rotifera MLKL, and mutations of some of the conserved residues in the α-helices significantly decreased the cell death-inducing ability of AvMLKL-1. These observations suggested that the pore-forming activity of MLKL-NTD was conserved in a manner that relied on the basic α-helix bundle, which had been evolutionarily selected in both invertebrates and vertebrates.

## 4. Materials and Methods

### 4.1. Sequence Collection

Sequence collection was performed as reported previously [41,42]. Briefly, 465 MLKL reference sequences were downloaded from NCBI orthologs and used as queries to search against the non-redundant database and specialized databases via BLASTP, with the E-value set as 1 × 10^−5^ to ensure, in part, accuracy [43]. The sequences were subjected to a conserved domain search (https://www.ncbi.nlm.nih.gov/cdd/), accessed on 23 June 2023 [44], and only those with intact MLKL-NT and CT domains were selected as MLKL. The protein sequences of the putative MLKL were further filtered based on the genomic location and aligned with Clustal Omega to remove duplicates [45].

### 4.2. Phylogeny and Synteny

Phylogenetic and syntenic analyses were performed as reported previously [41,42]. The phylogenetic tree of the major metazoan phyla was collected from the public knowledge-based Time Tree (http://timetree.org/), accessed on 1 October 2023 [46], and the phyla icons were downloaded from PhyloPic (http://www.phylopic.org/), accessed on 1 October 2023. Multiple sequence alignment was conducted with Clustal Omega [45]. The phylogenetic tree was constructed based on the maximum likelihood method using IQ-TREE 2 v.2.1.2 [47], with a bootstrap of 1000 replications. The JTT + F + R9 and VT + F + R3 substitution models were used based on the Bayesian information criterion (BIC) score for the metazoan and invertebrate trees, respectively, via ModelFinder [48]. The final presented trees were visualized with iTOL (https://itol.embl.de/) [49], accessed on 15 October 2023. Sequence identity was calculated with needleall. The conserved syntenic blocks near the MLKL loci in different species were based on the information from the genomic annotation of the NCBI database (http://www.ncbi.nlm.nih.gov/), accessed on 23 June 2023.

### 4.3. Transcriptome Analysis

The expression patterns of MLKL were based on the publicly available transcriptomes, i.e., *Homo sapiens* (PRJEB4337), *Mus musculus* (PRJNA66167), *Xenopus tropicalis* (E-MTAB-3726), *Gallus gallus* (E-MTAB-2797), *Perca fluviatilis* (PRJNA256973), *Astyanax mexicanus* (PRJNA285201), and *Pangasianodon hypophthalmus* (PRJNA256963).

### 4.4. Gene Cloning and Mutagenesis

The coding sequences of the MLKL variants and HsRIPK1/3 were synthesized by Tsingke Biotechnology (Nanjing, China) and Sangon Biotech (Shanghai, China). All the mutations were performed by using a Fast Mutagenesis System (Transgen Biotech, Beijing, China) according to the manufacturer’s instructions. The primers used for the mutagenesis are listed in the Supplemental Table S3.

### 4.5. Lactate Dehydrogenase (LDH) Assay

The MLKL-NTD coding sequences of all the examined species were cloned into pmCherry-N1 (Tsingke Biotechnology, Nanjing, China) at the HindIII and BamHI sites. The recombinant plasmids were used for transfection as follows. The HEK293T cells were seeded in 96-well plates (Costar, Corning, NY, USA) and incubated at 37 °C in 5% CO_2_ overnight. The cells were transfected with the indicated plasmid (0.1 μg per well) using Lipo3000 transfection reagent (Thermo Fisher Scientific, South Logan, UT, USA) according to the manufacturer’s instructions. After 24 h of transfection, the release of LDH from the cells was measured using the CytoTox 96 Non-Radioactive Cytotoxicity Assay (Promega, Leiden, The Netherlands) [50]. The percent LDH release was calculated using the following formula: 100 × (experimental sample − culture medium background)/(maximum LDH release − culture medium background).

### 4.6. Microscopy

The HEK293T cells were cultured and transfected as described above. After 24 h of transfection, the cells were stained with Sytox green and observed with a fluorescence microscope. For confocal microscopic examination of MLKL-induced necroptosis, the MLKL-NTD coding sequences of AvMLKL-1, ApMLKL, PmMLKL, CiMLKL, BfMLKL-2, and BlMLKL were cloned into the pCAGGS-Flag-C vector (Honor Gene, Changsha, China) at the EcoRI and NheI sites. The recombinant plasmids were used for transfection as follows. The HEK293T cells were plated in 35 mm culture dishes (Nest Scientific, Rahway, NJ, USA) and cultured overnight in DMEM supplemented with 10% FBS at 37 °C in an incubator containing 5% CO_2_. The cells were transfected with the above pCAGGS-Flag-C-based plasmids using Lipo3000 transfection reagent for 12 h. For cell membrane and nuclear staining, the cells were treated with Dil for 2 h and Sytox green for 10 min. The images were recorded with a Carl Zeiss LSM 900 confocal microscope (Carl Zeiss, Jena, Germany) as reported previously [51].

## 5. Conclusions

In this study, we demonstrated that MLKL homologues exist in both Deuterostomia and Protostomia, and that the Protostomia MLKL are more closely related to plant/fungi MLKL than to metazoan MLKL. Divergent evolution and copy number difference were observed between invertebrate and vertebrate MLKL. In vertebrates, the MLKL expression was detected abundantly in immune organs, suggesting an immune-related function of MLKL. The MLKL-NTD of Rotifera and three Deuterostomia phyla exhibited apparent necroptotic activity, which, in the case of Rotifera, required the existence of certain conserved residues of the NTD. These results added new insights into the evolution of MLKL in Metazoa and shed light on the biological activity of invertebrate MLKL.

## Data Availability

The data presented in this study can be accessed in the article/Supplemental Material.

## Supplementary Materials

The following supporting information can be downloaded at: https://www.mdpi.com/article/10.3390/ijms251910626/s1.
