# Supplementary material for: The Evolution and Biological Activity of Metazoan Mixed Lineage Kinase Domain-Like Protein (MLKL)
Source: Int J Mol Sci. 2024 Oct 2;25(19):10626. doi: 10.3390/ijms251910626 (PMC11476962; doi:10.3390/ijms251910626)
Supplement: Supplementary file 1 [file ijms-25-10626-s001.zip › ijms-3199020-supplementary.pdf]

Supplemental Figure S1. The phylogenetic analysis of invertebrate and plant/fungi MLKL. The phylogenetic tree was constructed with maximum likelihood analysis using the VT+F+R4 substitution model implemented in IQ-TREE 2 v.2.1.2. The tree is rooted at the midpoint, and the bootstraps are indicated.

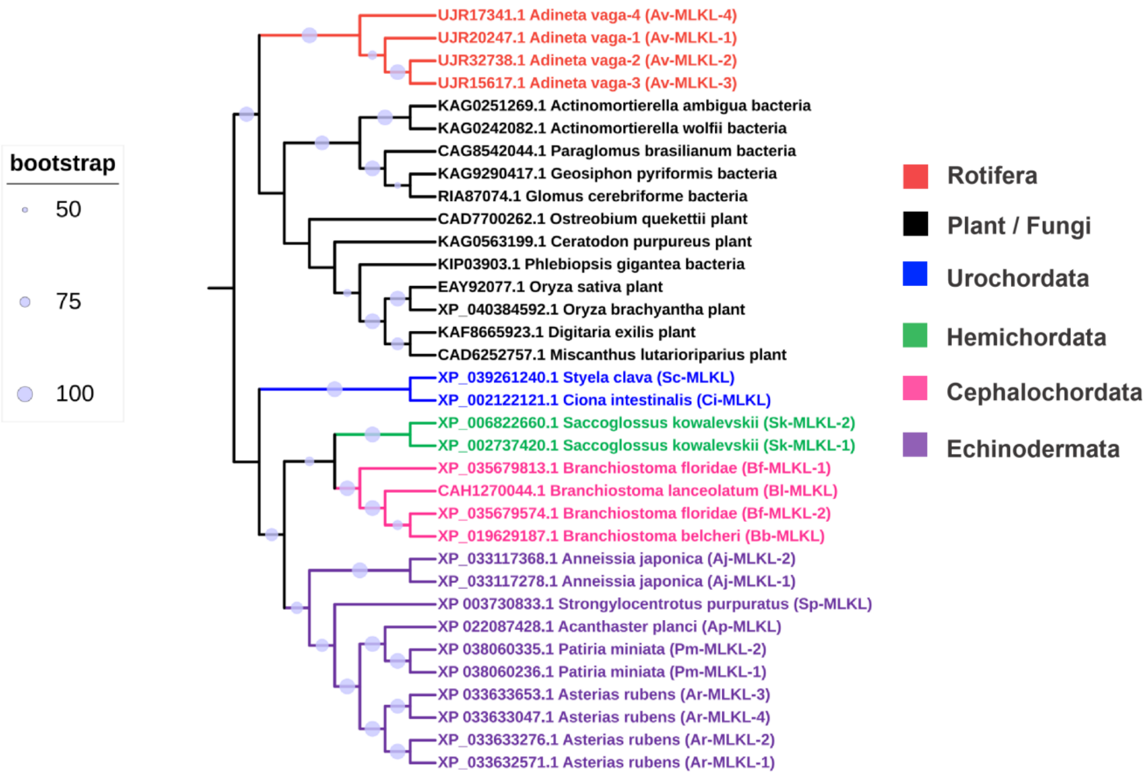

Supplemental Figure S2. Time-dependent cell death induced by MLKL. HEK293T cells were transfected with or without (control) AvMLKL-1-NTD **(A)**, CiMLKL-NTD **(B)**, BfMLKL-2-NTD **(C)**, BlMLKL-NTD **(D)**, ApMLKL-NTD **(E)**, and PmMLKL-1-NTD **(F)** for 8, 12, 16 and 24 h. LDH release was then determined. Data are the means ± SD of triplicate experiments. ***P < 0.001.

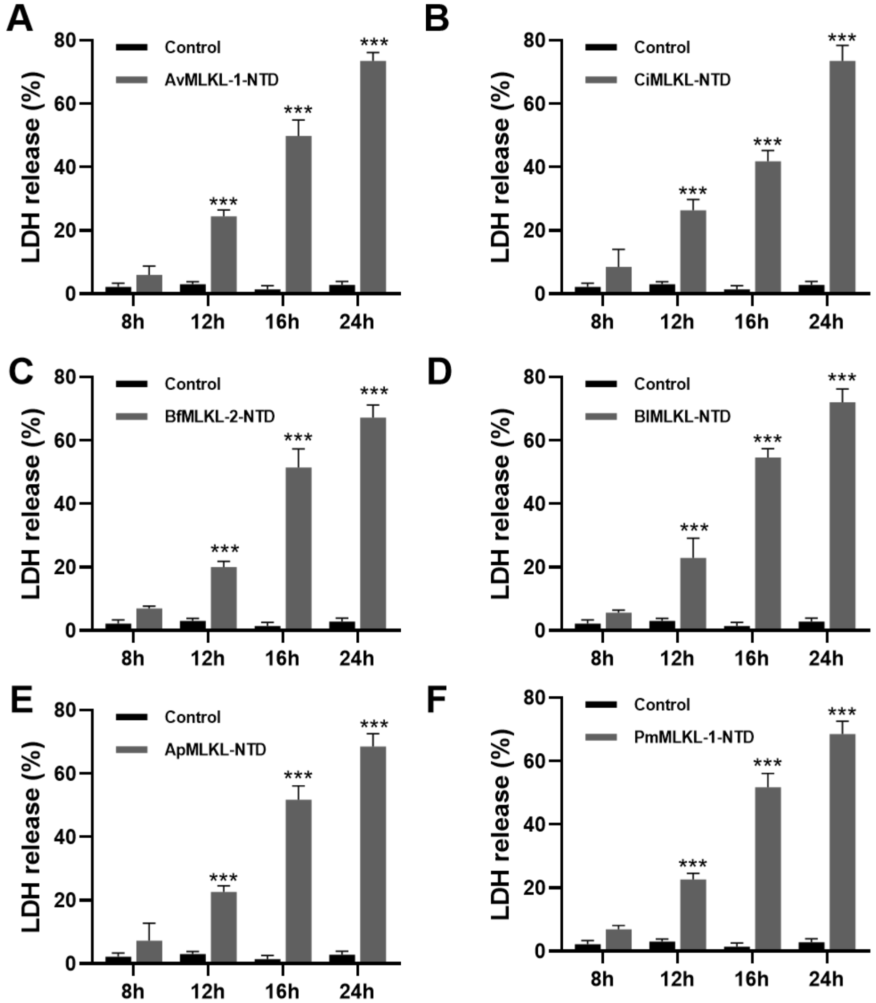

Supplemental Figure S3. The cytotoxicity of full-length AvMLKL. HEK293T cells were transfected with the backbone vector (control) or the vector expressing mCherry tagged AvMLKL-1/2/3/4 for 24 h. LDH release was then measured. Data are the means ± SD of triplicate experiments.

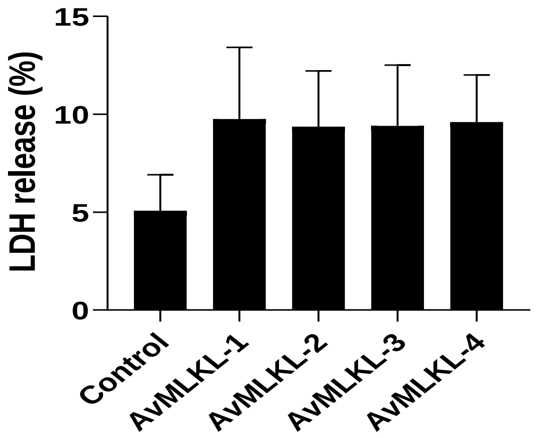

Supplemental Table S1. The accession numbers and species of the identified MLKL.

Clade	Accession number	Species
Invertebrate	XP_022087428.1	*Acanthaster planci*
	UJR15617.1	*Adineta vaga*
	UJR17341.1	*Adineta vaga*
	UJR20247.1	*Adineta vaga*
	UJR32738.1	*Adineta vaga*
	XP_033117368.1	*Anneissia japonica*
	XP_033117278.1	*Anneissia japonica*
	XP_033633047.1	*Asterias rubens*
	XP_033633276.1	*Asterias rubens*
	XP_033633653.1	*Asterias rubens*
	XP_033632571.1	*Asterias rubens*
	XP_019629187.1	*Branchiostoma belcheri*
	XP_035679813.1	*Branchiostoma floridae*
	XP_035679574.1	*Branchiostoma floridae*
	CAH1270044.1	*Branchiostoma lanceolatum*
	XP_002122121.1	*Ciona intestinalis*
	XP_038060335.1	*Patiria miniata*
	XP_038060236.1	*Patiria miniata*
	XP_002737420.1	*Saccoglossus kowalevskii*
	XP_006822660.1	*Saccoglossus kowalevskii*
	XP_003730833.1	*Strongylocentrotus purpuratus*
	XP_039261240.1	*Styela clava*
Cyclostomata	XP_032816483.1	*Petromyzon marinus*
Chondrichthyes	XP_032892006.1	*Amblyraja radiata*
	XP_007902865.1	*Callorhinchus milii*
	XP_041046767.1	*Carcharodon carcharias*
	XP_043563175.1	*Chiloscyllium plagiosum*
	GCC26452.1	*Chiloscyllium punctatum*
	XP_055504610.1	*Leucoraja erinacea*
	XP_051884266.1	*Pristis pectinata*
	XP_048461872.1	*Rhincodon typus*

	XP_038662909.1	*Scyliorhinus canicula*
	XP_038662910.1	*Scyliorhinus canicula*
	GCB78202.1	*Scyliorhinus torazame*
	GCB76521.1	*Scyliorhinus torazame*
	XP_048403148.1	*Stegostoma fasciatum*
	XP_048403146.1	*Stegostoma fasciatum*
Actinopterygii	XP_051808211.1	*Acanthochromis polyacanthus*
	XP_036964413.1	*Acanthopagrus latus*
	XP_033898255.2	*Acipenser ruthenus*
	XP_034784245.1	*Acipenser ruthenus*
	KAG9342006.1	*Albula glossodonta*
	KAI1904229.1	*Albula goreensis*
	KAJ8407923.1	*Aldrovandia affinis*
	XP_041966901.1	*Alosa sapidissima*
	KAF4091194.1	*Ameiurus melas*
	XP_023119167.2	*Amphiprion ocellaris*
	ROL46619.1	*Anabarilius grahami*
	XP_026220325.1	*Anabas testudineus*
	XP_031719497.1	*Anarrhichthys ocellatus*
	XP_035250777.1	*Anguilla anguilla*
	XP_054476216.1	*Anoplopoma fimbria*
	XP_030587542.1	*Archocentrus centrarchus*
	XP_030587658.1	*Archocentrus centrarchus*
	XP_026028222.1	*Astatotilapia calliptera*
	XP_026028224.1	*Astatotilapia calliptera*
	XP_026028346.1	*Astatotilapia calliptera*
	XP_022539844.2	*Astyanax mexicanus*
	XP_013886302.1	*Austrofundulus limnaeus*
	XP_029009933.1	*Betta splendens*
	XP_029009934.1	*Betta splendens*
	XP_020787832.1	*Boleophthalmus pectinirostris*
	XP_048885983.1	*Brienomyrus brachyistius*
	XP_026058901.1	*Carassius auratus*
	XP_026095188.1	*Carassius auratus*

	XP_052452892.1	*Carassius gibelio*
	XP_052399548.1	*Carassius gibelio*
	KAI4799414.1	*Chaenocephalus aceratus*
	KAF3706075.1	*Channa argus*
	XP_030622456.1	*Chanos chanos*
	XP_041650845.1	*Cheilinus undulatus*
	XP_041650846.1	*Cheilinus undulatus*
	XP_041795371.1	*Chelmon rostratus*
	XP_053361148.1	*Clarias gariepinus*
	KAF5892540.1	*Clarias magur*
	XP_031420999.1	*Clupea harengus*
	TKS73784.1	*Collichthys lucidus*
	TKS73880.1	*Collichthys lucidus*
	XP_036424303.1	*Colossoma macropomum*
	KAJ8252575.1	*Conger conger*
	KAJ8252574.1	*Conger conger*
	XP_041693032.1	*Coregonus clupeaformis*
	CAB1323444.1	*Coregonus sp. 'balchen'*
	XP_029290597.1	*Cottoperca gobio*
	XP_051740110.1	*Ctenopharyngodon idella*
	XP_034391522.1	*Cyclopterus lumpus*
	XP_008309816.1	*Cynoglossus semilaevis*
	XP_015253446.1	*Cyprinodon variegatus*
	XP_018951939.1	*Cyprinus carpio*
	XP_018954595.1	*Cyprinus carpio*
	XP_028814478.1	*Denticeps clupeoides*
	XP_051237873.1	*Dicentrarchus labrax*
	KAI9547375.1	*Dissostichus eleginoides*
	KAI9547379.1	*Dissostichus eleginoides*
	KAF3855360.1	*Dissostichus mawsoni*
	KAF3855373.1	*Dissostichus mawsoni*
	KAF3855354.1	*Dissostichus mawsoni*
	XP_029379770.1	*Echeneis naucrates*
	XP_026878528.2	*Electrophorus electricus*

	XP_049430797.1	*Epinephelus fuscoguttatus*
	XP_033490226.1	*Epinephelus lanceolatus*
	XP_049929362.1	*Epinephelus moara*
	XP_051787504.1	*Erpetoichthys calabaricus*
	XP_010903724.2	*Esox lucius*
	XP_032378872.1	*Etheostoma spectabile*
	XP_021169406.1	*Fundulus heteroclitus*
	XP_035985161.1	*Fundulus heteroclitus*
	XP_030222388.1	*Gadus morhua*
	XP_043979986.1	*Gambusia affinis*
	XP_040018983.1	*Gasterosteus aculeatus aculeatus*
	XP_047237074.1	*Girardinichthys multiradiatus*
	XP_034063985.1	*Gymnodraco acuticeps*
	XP_005946793.1	*Haplochromis burtoni*
	XP_042072668.1	*Haplochromis burtoni*
	XP_042082328.1	*Haplochromis burtoni*
	KAG7329377.1	*Hemibagrus wyckioides*
	XP_034443287.1	*Hippoglossus hippoglossus*
	XP_034443289.1	*Hippoglossus hippoglossus*
	XP_047195977.1	*Hippoglossus stenolepis*
	XP_046890836.1	*Hypomesus transpacificus*
	XP_053487404.1	*Ictalurus furcatus*
	XP_017330243.1	*Ictalurus punctatus*
	XP_037834251.1	*Kryptolebias marmoratus*
	XP_037834252.1	*Kryptolebias marmoratus*
	XP_050954907.1	*Labeo rohita*
	XP_020490581.1	*Labrus bergylta*
	XP_010748607.2	*Larimichthys crocea*
	XP_018516011.1	*Lates calcarifer*
	XP_050929236.1	*Lates calcarifer*
	XP_015223778.1	*Lepisosteus oculatus*
	TNN38732.1	*Liparis tanakae*
	XP_026167124.1	*Mastacembelus armatus*
	XP_004566214.1	*Maylandia zebra*

	XP_014266785.2	*Maylandia zebra*
	XP_024655821.1	*Maylandia zebra*
	XP_048014940.1	*Megalobrama amblycephala*
	XP_036391352.1	*Megalops cyprinoides*
	XP_041854071.1	*Melanotaenia boesemani*
	CAG5896330.1	*Menidia menidia*
	XP_045894606.1	*Micropterus dolomieu*
	XP_038562675.1	*Micropterus salmoides*
	XP_055048703.1	*Misgurnus anguillicaudatus*
	XP_020443682.1	*Monopterus albus*
	XP_035520236.1	*Morone saxatilis*
	XP_047451261.1	*Mugil cephalus*
	XP_047451242.1	*Mugil cephalus*
	KAJ3580698.1	*Muraenolepis orangiensis*
	XP_029908639.1	*Myripristis murdjan*
	XP_051545525.1	*Myxocyprinus asiaticus*
	XP_037538440.1	*Nematolebias whitei*
	XP_006804005.1	*Neolamprologus brichardi*
	XP_035770061.1	*Neolamprologus brichardi*
	XP_015796673.1	*Nothobranchius furzeri*
	XP_034535100.1	*Notolabrus celidotus*
	XP_010766222.1	*Notothenia coriiceps*
	XP_046208464.1	*Oncorhynchus gorbuscha*
	XP_046210725.1	*Oncorhynchus gorbuscha*
	XP_035646648.1	*Oncorhynchus keta*
	XP_035593624.1	*Oncorhynchus keta*
	XP_020347972.1	*Oncorhynchus kisutch*
	XP_031657216.1	*Oncorhynchus kisutch*
	XP_036808320.1	*Oncorhynchus mykiss*
	XP_021454554.1	*Oncorhynchus mykiss*
	XP_021423147.2	*Oncorhynchus mykiss*
	XP_029541761.1	*Oncorhynchus nerka*
	XP_029524273.1	*Oncorhynchus nerka*
	XP_024244586.1	*Oncorhynchus tshawytscha*

	XP_024234547.1	*Oncorhynchus tshawytscha*
	KAF4095360.1	*Onychostoma macrolepis*
	XP_031601942.1	*Oreochromis aureus*
	XP_031589121.2	*Oreochromis aureus*
	XP_003439595.1	*Oreochromis niloticus*
	XP_019216476.1	*Oreochromis niloticus*
	XP_019216477.1	*Oreochromis niloticus*
	RVE65523.1	*Oryzias javanicus*
	RVE65525.1	*Oryzias javanicus*
	RVE65524.1	*Oryzias javanicus*
	XP_004075126.1	*Oryzias latipes*
	XP_011480342.1	*Oryzias latipes*
	XP_024155204.1	*Oryzias melastigma*
	XP_036070382.1	*Oryzias melastigma*
	MCI4380184.1	*Pangasianodon gigas*
	XP_026773361.2	*Pangasianodon hypophthalmus*
	MCJ8734293.1	*Pangasius djambal*
	XP_019934153.1	*Paralichthys olivaceus*
	XP_028288043.1	*Parambassis ranga*
	XP_028270238.1	*Parambassis ranga*
	XP_023701337.1	*Paramormyrops kingsleyae*
	XP_028441066.1	*Perca flavescens*
	XP_028440177.1	*Perca flavescens*
	XP_039665593.1	*Perca fluviatilis*
	XP_039665591.1	*Perca fluviatilis*
	XP_033824481.1	*Periophthalmus magnuspinnatus*
	XP_039519076.1	*Pimephales promelas*
	XP_042352442.1	*Plectropomus leopardus*
	XP_053283395.1	*Pleuronectes platessa*
	XP_007560561.1	*Poecilia formosa*
	XP_007551461.1	*Poecilia formosa*
	XP_014901641.1	*Poecilia latipinna*
	XP_014866740.1	*Poecilia mexicana*
	XP_014829976.1	*Poecilia mexicana*

	XP_008417620.1	*Poecilia reticulata*
	XP_008436190.1	*Poecilia reticulata*
	XP_054912140.1	*Poeciliopsis prolifica*
	KAJ4921855.1	*Pogonophryne albipinna*
	KAJ4935746.1	*Pogonophryne albipinna*
	XP_041077011.1	*Polyodon spathula*
	XP_041125449.1	*Polyodon spathula*
	XP_039619540.1	*Polypterus senegalus*
	KAI4891344.1	*Prochilodus magdalenae*
	XP_033941170.1	*Pseudochaenichthys georgianus*
	XP_005745215.1	*Pundamilia nyererei*
	XP_037308327.1	*Pungitius pungitius*
	XP_043083170.1	*Puntigrus tetrazona*
	XP_043083312.1	*Puntigrus tetrazona*
	XP_043084145.1	*Puntigrus tetrazona*
	XP_017545667.1	*Pygocentrus nattereri*
	XP_029952568.1	*Salarias fasciatus*
	XP_029953003.1	*Salarias fasciatus*
	NP_001133765.1	*Salmo salar*
	XP_013980000.2	*Salmo salar*
	XP_045562301.1	*Salmo salar*
	XP_029613950.1	*Salmo trutta*
	XP_029607336.1	*Salmo trutta*
	XP_029613953.1	*Salmo trutta*
	XP_023840873.1	*Salvelinus alpinus*
	XP_023854605.1	*Salvelinus alpinus*
	XP_023840874.1	*Salvelinus alpinus*
	XP_055790556.1	*Salvelinus fontinalis*
	XP_055790557.1	*Salvelinus fontinalis*
	XP_038873216.1	*Salvelinus namaycush*
	XP_038873211.1	*Salvelinus namaycush*
	XP_038854585.1	*Salvelinus namaycush*
	XP_031135715.1	*Sander lucioperca*
	XP_046251025.1	*Scatophagus argus*

	XP_018618691.1	*Scleropages formosus*
	XP_053175576.1	*Scomber japonicus*
	XP_035498615.2	*Scophthalmus maximus*
	KAI3371461.1	*Scortum barcoo*
	XP_037622275.1	*Sebastes umbrosus*
	XP_022620289.1	*Seriola dumerili*
	XP_023276148.1	*Seriola lalandi dorsalis*
	KAI5617542.1	*Silurus asotus*
	XP_046706457.1	*Silurus meridionalis*
	XP_039902930.1	*Simochromis diagramma*
	XP_039894668.1	*Simochromis diagramma*
	XP_039894670.1	*Simochromis diagramma*
	XP_044049928.1	*Siniperca chuatsi*
	XP_016354688.1	*Sinocyclocheilus anshuiensis*
	XP_016103042.1	*Sinocyclocheilus grahami*
	XP_016367085.1	*Sinocyclocheilus rhinocerous*
	XP_043892867.1	*Solea senegalensis*
	XP_030282795.1	*Sparus aurata*
	XP_030282796.1	*Sparus aurata*
	XP_030282794.1	*Sparus aurata*
	XP_029991575.1	*Sphaeramia orbicularis*
	XP_008303596.1	*Stegastes partitus*
	KAJ8343377.1	*Synaphobranchus kaupii*
	XP_053741524.1	*Synchiropus splendidus*
	XP_026990918.1	*Tachysurus fulvidraco*
	CAG01083.1	*Tetraodon nigroviridis*
	XP_034033227.1	*Thalassophryne amazonica*
	XP_044211856.1	*Thunnus albacares*
	XP_044211855.1	*Thunnus albacares*
	XP_042266757.1	*Thunnus maccoyii*
	XP_042266756.1	*Thunnus maccoyii*
	XP_042266755.1	*Thunnus maccoyii*
	XP_040895169.1	*Toxotes jaculatrix*
	XP_033982799.1	*Trematomus bernacchii*

	XP_033982768.1	*Trematomus bernacchii*
	XP_033982822.1	*Trematomus bernacchii*
	XP_033982769.1	*Trematomus bernacchii*
	KAI7811627.1	*Triplophysa rosa*
	KAA0724109.1	*Triplophysa tibetana*
	XP_039990002.1	*Xiphias gladius*
	XP_027885747.1	*Xiphophorus couchianus*
	XP_027885746.1	*Xiphophorus couchianus*
	XP_032429535.1	*Xiphophorus hellerii*
	XP_032429534.1	*Xiphophorus hellerii*
	XP_032403263.1	*Xiphophorus hellerii*
	XP_005807411.1	*Xiphophorus maculatus*
	XP_023203021.1	*Xiphophorus maculatus*
	XP_005808747.2	*Xiphophorus maculatus*
	XP_051975891.1	*Xyrauchen texanus*
Coelacanthimorpha	XP_006010420.1	*Latimeria chalumnae*
Dipnomorpha	XP_043937895.1	*Protopterus annectens*
	XP_043937894.1	*Protopterus annectens*
Amphibia	XP_053558124.1	*Bombina bombina*
	XP_053558146.1	*Bombina bombina*
	XP_040265300.1	*Bufo bufo*
	XP_040265299.1	*Bufo bufo*
	XP_044125886.1	*Bufo gargarizans*
	KAG9474781.1	*Eleutherodactylus coqui*
	KAG8562425.1	*Engystomops pustulosus*
	KAG8562426.1	*Engystomops pustulosus*
	KAG8437249.1	*Hymenochirus boettgeri*
	XP_030059485.1	*Microcaecilia unicolor*
	XP_018428895.1	*Nanorana parkeri*
	KAJ1081730.1	*Pleurodeles waltl*
	XP_040185287.1	*Rana temporaria*
	XP_040185288.1	*Rana temporaria*
	XP_040185286.1	*Rana temporaria*
	XP_029464741.1	*Rhinatrema bivittatum*

	XP_053305489.1	*Spea bombifrons*
	XP_053305491.1	*Spea bombifrons*
	XP_018113556.1	*Xenopus laevis*
	XP_041446179.1	*Xenopus laevis*
	KAE8609144.1	*Xenopus tropicalis*
Squamata	XP_003229262.1	*Anolis carolinensis*
	XP_039209654.1	*Crotalus tigris*
	XP_054856660.1	*Eublepharis macularius*
	XP_015285204.1	*Gekko japonicus*
	XP_015285205.1	*Gekko japonicus*
	XP_053127337.1	*Hemicordylus capensis*
	XP_033013311.1	*Lacerta agilis*
	KAJ6666206.1	*Lerista edwardsae*
	KAG8143859.1	*Naja naja*
	XP_026535903.1	*Notechis scutatus*
	XP_034281300.1	*Pantherophis guttatus*
	KAJ7312110.1	*Phrynocephalus forsythii*
	XP_028595160.1	*Podarcis muralis*
	XP_053255240.1	*Podarcis raffonei*
	XP_020653567.1	*Pogona vitticeps*
	XP_015671356.1	*Protobothrops mucrosquamatus*
	XP_026570185.1	*Pseudonaja textilis*
	XP_025027125.1	*Python bivittatus*
	XP_048371781.1	*Sphaerodactylus townsendi*
	XP_032087076.1	*Thamnophis elegans*
	XP_044290260.1	*Varanus komodoensis*
	XP_034973777.1	*Zootoca vivipara*
	XP_034973776.1	*Zootoca vivipara*
Testudines	XP_048673050.1	*Caretta caretta*
	XP_007071298.3	*Chelonia mydas*
	KAG6923403.1	*Chelydra serpentina*
	XP_008173597.1	*Chrysemys picta bellii*
	XP_038224372.1	*Dermochelys coriacea*
	XP_030438000.1	*Gopherus evgoodei*

	XP_030437999.1	*Gopherus evgoodei*
	XP_050778367.1	*Gopherus flavomarginatus*
	XP_053904499.1	*Malaclemys terrapin pileata*
	XP_053904498.1	*Malaclemys terrapin pileata*
	XP_044843708.1	*Mauremys mutica*
	XP_039358730.1	*Mauremys reevesii*
	XP_006115190.1	*Pelodiscus sinensis*
	XP_006115189.1	*Pelodiscus sinensis*
	TFJ98604.1	*Platysternon megacephalum*
	XP_034644557.1	*Trachemys scripta elegans*
Crocodilia	XP_019391159.1	*Crocodylus porosus*
	XP_019391158.1	*Crocodylus porosus*
Aves	XP_009071913.1	*Acanthisitta chloris*
	XP_049661767.1	*Accipiter gentilis*
	NWZ63868.1	*Acrocephalus arundinaceus*
	NWH89223.1	*Aegithalos caudatus*
	NWX19048.1	*Aegotheles bennettii*
	XP_054497003.1	*Agelaius phoeniceus*
	KAI6077537.1	*Aix galericulata*
	NXQ36492.1	*Alaudala cheleensis*
	NWX68780.1	*Alca torda*
	NXC55507.1	*Aleadryas rufinucha*
	NXL95836.1	*Alectura lathami*
	NXW84039.1	*Alopecoenas beccarii*
	NXK78401.1	*Amazona guildingii*
	XP_005009126.2	*Anas platyrhynchos*
	NXC77814.1	*Anhinga anhinga*
	NXT86689.1	*Anhinga rufa*
	XP_047934653.1	*Anser cygnoides*
	NXI71656.1	*Anseranas semipalmata*
	NXQ60447.1	*Anthoscopus minutus*
	XP_010167376.1	*Antrostomus carolinensis*
	XP_009869845.1	*Apaloderma vittatum*
	NWY10606.1	*Aphelocoma coerulescens*

	XP_009289133.1	*Aptenodytes forsteri*
	KAF1651734.1	*Aptenodytes patagonicus*
	XP_013808065.1	*Apteryx mantelli mantelli*
	XP_025944378.1	*Apteryx rowi*
	XP_051485215.1	*Apus apus*
	XP_029881668.1	*Aquila chrysaetos chrysaetos*
	NXO53816.1	*Aramus guarauna*
	NXE26316.1	*Ardeotis kori*
	NXK21686.1	*Arenaria interpres*
	NWZ23269.1	*Asarcornis scutulata*
	XP_026712431.1	*Athene cunicularia*
	NXV70792.1	*Atlantisia rogersi*
	NXY15751.1	*Atrichornis clamosus*
	XP_032051654.1	*Aythya fuligula*
	NXS39417.1	*Balaeniceps rex*
	XP_010297767.1	*Balearica regulorum gibbericeps*
	POI23336.1	*Bambusicola thoracicus*
	NXG68879.1	*Baryphthengus martii*
	NXN87203.1	*Bombycilla garrulus*
	NWZ34916.1	*Brachypodius atriceps*
	NXS61647.1	*Brachypteracias leptosomus*
	NXH22214.1	*Bucco capensis*
	XP_010138752.1	*Buceros rhinoceros silvestris*
	NWR60098.1	*Bucorvus abyssinicus*
	NXU07388.1	*Buphagus erythrorhynchus*
	NWQ96005.1	*Burhinus bistriatus*
	NXE64469.1	*Calcarius ornatus*
	XP_014816246.1	*Calidris pugnax*
	NXY55924.1	*Callaeas wilsoni*
	NWX03590.1	*Caloenas nicobarica*
	NXV86057.1	*Calonectris borealis*
	XP_008498941.1	*Calypte anna*
	NWI54002.1	*Calyptomena viridis*
	XP_030812104.1	*Camarhynchus parvulus*

	NXC35378.1	*Campylorhamphus procurvoides*
	NWT34133.1	*Cardinalis cardinalis*
	XP_009708334.1	*Cariama cristata*
	NXE55541.1	*Casuarius casuarius*
	KFP55267.1	*Cathartes aura*
	NXQ46972.1	*Catharus fuscescens*
	XP_032925691.1	*Catharus ustulatus*
	XP_042690723.1	*Centrocercus urophasianus*
	NXX95729.1	*Centropus bengalensis*
	NWR79167.1	*Centropus unirufus*
	NWU12183.1	*Cephalopterus ornatus*
	NXV24504.1	*Cepphus grylle*
	NXC91006.1	*Cercotrichas coryphoeus*
	NXO99465.1	*Certhia brachydactyla*
	NXC97282.1	*Certhia familiaris*
	NXU97921.1	*Cettia cetti*
	NXY51165.1	*Ceuthmochares aereus*
	NXT62981.1	*Chaetops frenatus*
	NXD99403.1	*Chaetorhynchus papuensis*
	XP_009996615.1	*Chaetura pelagica*
	XP_009881458.1	*Charadrius vociferus*
	NXK45989.1	*Chauna torquata*
	NWY56293.1	*Chionis minor*
	XP_032557072.1	*Chiroxiphia lanceolata*
	XP_010124379.1	*Chlamydotis macqueenii*
	RLW01253.1	*Chloebia gouldiae*
	NXI50112.1	*Chloroceryle aenea*
	NXP60337.1	*Chloropsis cyanopogon*
	NXL65993.1	*Chordeiles acutipennis*
	NWT46037.1	*Chroicocephalus maculipennis*
	NWS63300.1	*Chunga burmeisteri*
	NXF59834.1	*Ciccaba nigrolineata*
	NXJ38473.1	*Ciconia maguari*
	NXR25679.1	*Cinclus mexicanus*

	NXW24955.1	*Circaetus pectoralis*
	NXO23932.1	*Cisticola juncidis*
	NWW77715.1	*Climacteris rufus*
	NXB00434.1	*Cnemophilus loriae*
	NXE83682.1	*Cochlearius cochlearius*
	OXB74078.1	*Colinus virginianus*
	XP_010206138.1	*Colius striatus*
	XP_005504837.1	*Columba livia*
	NXD39182.1	*Copsychus sechellarum*
	XP_027502931.1	*Corapipo altera*
	XP_008637110.1	*Corvus brachyrhynchos*
	XP_031977878.1	*Corvus moneduloides*
	NXC22642.1	*Corythaeola cristata*
	NXJ90331.1	*Corythaixoides concolor*
	XP_015729096.1	*Coturnix japonica*
	NWS76185.1	*Crotophaga sulcirostris*
	XP_009568267.2	*Cuculus canorus*
	XP_023790715.1	*Cyanistes caeruleus*
	XP_035399361.1	*Cygnus atratus*
	XP_040427259.1	*Cygnus olor*
	NWV45995.1	*Daphoenositta chrysoptera*
	NWV83919.1	*Dasyornis broadbenti*
	NXH40213.1	*Dicaeum eximium*
	NXJ28328.1	*Dicrurus megarhynchus*
	NXB76340.1	*Donacobius atricapilla*
	XP_025963144.1	*Dromaius novaehollandiae*
	NWU51275.1	*Dromas ardeola*
	NXU40999.1	*Drymodes brunneopygia*
	XP_009904197.2	*Dryobates pubescens*
	NWI77040.1	*Dryoscopus gambensis*
	NXH92761.1	*Edolisoma coerulescens*
	NXD25130.1	*Elachura formosa*
	NWR19672.1	*Emberiza fucata*
	XP_027751489.1	*Empidonax traillii*

	NWY72783.1	*Erithacus rubecula*
	NXS77811.1	*Erpornis zantholeuca*
	NXF97675.1	*Eubucco bourcierii*
	KAF1652795.1	*Eudyptes chrysocome*
	KAF1592760.1	*Eudyptes moseleyi*
	KAF1589585.1	*Eudyptes pachyrhynchus*
	KAF1561159.1	*Eudyptes robustus*
	KAF1537714.1	*Eudyptes schlegeli*
	KAF1513037.1	*Eudyptes sclateri*
	KAF1504752.1	*Eudyptula minor*
	KAF1496362.1	*Eudyptula novaehollandiae*
	NXB39045.1	*Eulacestoma nigropectus*
	XP_010152813.1	*Eurypyga helias*
	NXW57976.1	*Eurystomus gularis*
	XP_005446870.1	*Falco cherrug*
	XP_040471106.1	*Falco naumanni*
	XP_005233697.2	*Falco peregrinus*
	NWW19338.1	*Falcunculus frontatus*
	XP_005052681.1	*Ficedula albicollis*
	NXK87206.1	*Formicarius rufipectus*
	NWH50389.1	*Fregata magnificens*
	NXW03097.1	*Fregetta grallaria*
	XP_009583474.1	*Fulmarus glacialis*
	NWR84513.1	*Furnarius figulus*
	NXI34959.1	*Galbula dea*
	XP_040536926.1	*Gallus gallus*
	XP_009819422.1	*Gavia stellata*
	NWH58036.1	*Geococcyx californianus*
	XP_005422873.1	*Geospiza fortis*
	NXY76209.1	*Glareola pratincola*
	NXL38146.1	*Glaucidium brasilianum*
	NXG22678.1	*Grallaria varia*
	NWV36132.1	*Grantiella picta*
	XP_054696938.1	*Grus americana*

	XP_050760204.1	*Gymnogyps californianus*
	NXM51496.1	*Gymnorhina tibicen*
	NXD84372.1	*Halcyon senegalensis*
	XP_009921368.1	*Haliaeetus albicilla*
	XP_052652585.1	*Harpia harpyja*
	NXP47492.1	*Heliornis fulica*
	NXG63149.1	*Hemiprocne comata*
	NXK09864.1	*Herpetotheres cachinnans*
	NXN70717.1	*Himantopus himantopus*
	NXR52289.1	*Hippolais icterina*
	XP_039931492.1	*Hirundo rustica*
	NXU69254.1	*Horornis vulcanius*
	NWU34133.1	*Hylia prasina*
	NXR91646.1	*Hypocryptadius cinnamomeus*
	NXA17671.1	*Ibidorhyncha struthersii*
	NWW62309.1	*Ifrita kowaldi*
	NXM53347.1	*Illadopsis cleaveri*
	XP_054245855.1	*Indicator indicator*
	NXN06449.1	*Indicator maculatus*
	NXI15080.1	*Irena cyanogastra*
	NXT03340.1	*Jacana jacana*
	XP_042742047.1	*Lagopus leucura*
	XP_048814155.1	*Lagopus muta*
	KAI1235472.1	*Lamprotornis superbus*
	NWT86071.1	*Lanius ludovicianus*
	NXP37575.1	*Leiothrix lutea*
	XP_017679931.1	*Lepidothrix coronata*
	NXL81817.1	*Leptocoma aspasia*
	XP_009949956.1	*Leptosomus discolor*
	NXB54342.1	*Leucopsar rothschildi*
	PKU32125.1	*Limosa lapponica baueri*
	NXO36691.1	*Locustella ochotensis*
	XP_031361700.1	*Lonchura striata domestica*
	NXE13703.1	*Lophotis ruficrista*

	NWY90525.1	*Loxia curvirostra*
	NXH04795.1	*Loxia leucoptera*
	NWV87795.1	*Machaerirhynchus nigripectus*
	NWV60797.1	*Malurus elegans*
	XP_051627299.1	*Manacus candei*
	XP_029821344.1	*Manacus vitellinus*
	KAF1500901.1	*Megadyptes antipodes antipodes*
	NXA90646.1	*Melanocharis versteri*
	XP_010716210.1	*Meleagris gallopavo*
	XP_033928875.1	*Melopsittacus undulatus*
	KAF2978880.1	*Melospiza melodia maxima*
	XP_054125531.1	*Melozone crissalis*
	NXE98055.1	*Menura novaehollandiae*
	XP_008947666.1	*Merops nubicus*
	NXL12404.1	*Mesembrinibis cayennensis*
	XP_010189818.1	*Mesitornis unicolor*
	NWT03653.1	*Mionectes macconnelli*
	NXA65745.1	*Mohoua ochrocephala*
	XP_036246407.1	*Molothrus ater*
	NWR97596.1	*Motacilla alba*
	XP_038004224.1	*Motacilla alba alba*
	NXH24015.1	*Myiagra hebetior*
	XP_050167337.1	*Myiozetetes cayanensis*
	NXS25483.1	*Mystacornis crossleyi*
	NXS14158.1	*Neodrepanis coruscans*
	XP_027554090.1	*Neopelma chrysocephalum*
	NWQ67422.1	*Neopipo cinnamomea*
	NXA01056.1	*Nesospiza acunhae*
	XP_010013466.1	*Nestor notabilis*
	NXX27986.1	*Nicator chloris*
	XP_009468805.1	*Nipponia nippon*
	NXA53044.1	*Nothocercus julius*
	NXD15453.1	*Nothocercus nigrocapillus*
	NWY01799.1	*Nothoprocta ornata*

	NWX92187.1	*Nothoprocta pentlandii*
	XP_025890122.1	*Nothoprocta perdicaria*
	NWX32060.1	*Notiomystis cincta*
	XP_021264581.1	*Numida meleagris*
	NXF37431.1	*Nyctibius bracteatus*
	NXQ81020.1	*Nyctibius grandis*
	NXN32119.1	*Nycticryphes semicollaris*
	NXW49458.1	*Nyctiprogne leucopyga*
	NXF53577.1	*Oceanites oceanicus*
	NXH71384.1	*Oceanodroma tethys*
	NXJ07023.1	*Odontophorus gujanensis*
	NXM90431.1	*Oenanthe oenanthe*
	NWU78062.1	*Onychorhynchus coronatus*
	XP_041256707.1	*Onychostruthus taczanowskii*
	XP_009937264.1	*Opisthocomus hoazin*
	NWW05308.1	*Oreocharis arfaki*
	NXU78005.1	*Oreotrochilus melanogaster*
	NWV22421.1	*Origma solitaria*
	NXO16323.1	*Oriolus oriolus*
	NXC00945.1	*Orthonyx spaldingii*
	NXS04819.1	*Oxylabes madagascariensis*
	NXM26785.1	*Oxyruncus cristatus*
	XP_035192831.1	*Oxyura jamaicensis*
	NXH95400.1	*Pachycephala philippinensis*
	NWS12007.1	*Pachyramphus minor*
	NXS70610.1	*Pandion haliaetus*
	NWW36854.1	*Panurus biarmicus*
	NXU19400.1	*Pardalotus punctatus*
	XP_015495691.1	*Parus major*
	XP_039577416.1	*Passer montanus*
	NXP89773.1	*Passerina amoena*
	OPJ78114.1	*Patagioenas fasciata monilis*
	NWW45709.1	*Pedionomus torquatus*
	NXT34180.1	*Pelecanoides urinatrix*

	XP_009476634.1	*Pelecanus crispus*
	NXC41255.1	*Penelope pileata*
	NXQ10198.1	*Peucedramus taeniatus*
	XP_010294720.1	*Phaethon lepturus*
	NXW35401.1	*Phaetusa simplex*
	NXO68574.1	*Phainopepla nitens*
	XP_009501988.1	*Phalacrocorax carbo*
	XP_031470981.1	*Phasianus colchicus*
	NWY29668.1	*Pheucticus melanocephalus*
	KFQ77564.1	*Phoenicopterus ruber ruber*
	NWH70697.1	*Piaya cayana*
	NWI42543.1	*Picathartes gymnocephalus*
	XP_039236767.1	*Pipra filicauda*
	NXK40243.1	*Piprites chloris*
	KAJ7417821.1	*Pitangus sulphuratus*
	NWI93323.1	*Pitta sordida*
	NWU24981.1	*Platysteira castanea*
	NXM21906.1	*Ploceus nigricollis*
	NXT50698.1	*Pluvianellus socialis*
	NXX17818.1	*Podargus strigoides*
	KFZ58853.1	*Podiceps cristatus*
	NXL46301.1	*Podilymbus podiceps*
	NWZ80571.1	*Poecile atricapillus*
	NWS25050.1	*Polioptila caerulea*
	NXY35743.1	*Pomatorhinus ruficollis*
	NXS37357.1	*Pomatostomus ruficeps*
	NWS50606.1	*Probosciger aterrimus*
	NWX59261.1	*Promerops cafer*
	NXT04535.1	*Prunella fulvescens*
	NWT74673.1	*Prunella himalayana*
	XP_005526484.1	*Pseudopodoces humilis*
	NXG46798.1	*Psilopogon haemacephalus*
	NXI91968.1	*Psophia crepitans*
	NWU64165.1	*Pterocles burchelli*

	XP_010085282.1	*Pterocles gutturalis*
	NXY08965.1	*Pteruthius melanotis*
	NWV03978.1	*Ptilonorhynchus violaceus*
	NXR81274.1	*Pycnonotus jocosus*
	XP_009323641.1	*Pygoscelis adeliae*
	KAF1490135.1	*Pygoscelis antarcticus*
	KAF1676905.1	*Pygoscelis papua*
	XP_041314911.1	*Pyrgilauda ruficollis*
	NXQ68260.1	*Quiscalus mexicanus*
	NXP70246.1	*Ramphastos sulfuratus*
	NWR43408.1	*Regulus satrapa*
	NXH59128.1	*Rhabdornis inornatus*
	NXR68938.1	*Rhadina sibilatrix*
	NXB13898.1	*Rhagologus leucostigma*
	NXO01353.1	*Rhinopomastus cyanomelas*
	NXN44796.1	*Rhinoptilus africanus*
	NXI78746.1	*Rhipidura dahli*
	NXF16831.1	*Rhodinocichla rosea*
	NWW91504.1	*Rhynochetos jubatus*
	XP_054057223.1	*Rissa tridactyla*
	NXJ65306.1	*Rostratula benghalensis*
	NXN49522.1	*Rynchops niger*
	NXQ93377.1	*Sagittarius serpentarius*
	NXG03727.1	*Sakesphorus luctuosus*
	NXA07728.1	*Sapayoa aenigma*
	NXF80413.1	*Sclerurus mexicanus*
	NXX54046.1	*Scopus umbretta*
	NXP18272.1	*Scytalopus superciliaris*
	NXR15852.1	*Semnornis frantzii*
	NXM76473.1	*Serilophus lunatus*
	XP_009088921.2	*Serinus canaria*
	NXL16362.1	*Setophaga kirtlandii*
	NWR09496.1	*Sinosuthora webbiana*
	NXO82725.1	*Sitta europaea*

	NXF11603.1	*Smithornis capensis*
	KAF1398355.1	*Spheniscus magellanicus*
	KAF1413650.1	*Spheniscus mendiculus*
	NXJ53230.1	*Spizaetus tyrannus*
	NWX40813.1	*Steatornis caripensis*
	NXG89033.1	*Stercorarius parasiticus*
	NXI25817.1	*Sterrhoptilus dennistouni*
	XP_030326489.1	*Strigops habroptila*
	NXB55367.1	*Struthidea cinerea*
	XP_009678059.1	*Struthio camelus australis*
	XP_014736338.1	*Sturnus vulgaris*
	NWI33467.1	*Sula dactylatra*
	NWY37801.1	*Sylvia atricapilla*
	NXM99343.1	*Sylvia borin*
	NXK68322.1	*Sylvietta virens*
	NXT25578.1	*Syrrhaptes paradoxus*
	NWR34660.1	*Tachuris rubrigastra*
	XP_030138069.3	*Taeniopygia guttata*
	XP_009988492.1	*Tauraco erythrolophus*
	NXU31473.1	*Thalassarche chlororhynchos*
	NXP08731.1	*Thinocorus orbignyianus*
	NXA74889.1	*Thryothorus ludovicianus*
	NWI01085.1	*Tichodroma muraria*
	XP_010209292.1	*Tinamus guttatus*
	NWI71654.1	*Todus mexicanus*
	NWS79819.1	*Toxostoma redivivum*
	NXX44119.1	*Tricholaema leucomelas*
	NXJ83256.1	*Trogon melanurus*
	NXU57304.1	*Turnix velox*
	XP_052541261.1	*Tympanuchus pallidicinctus*
	NXM05079.1	*Tyrannus savana*
	XP_009968110.3	*Tyto alba*
	NWU95972.1	*Upupa epops*
	NXV49806.1	*Uria aalge*

	NXX79540.1	*Urocolius indicus*
	NWU01011.1	*Urocynchramus pylzowi*
	XP_053808892.1	*Vidua chalybeata*
	XP_053843433.1	*Vidua macroura*
	NWT08902.1	*Vireo altiloquus*
	NXT79437.1	*Zapornia atra*
	XP_026651481.1	*Zonotrichia albicollis*
	NXR30233.1	*Zosterops hypoxanthus*
Mammalia	XP_051024917.1	*Acomys russatus*
	XP_012292951.1	*Aotus nancymaae*
	XP_052023027.1	*Apodemus sylvaticus*
	XP_053522684.1	*Artibeus jamaicensis*
	XP_034378636.1	*Arvicanthis niloticus*
	XP_038168744.1	*Arvicola amphibius*
	KAB0389989.1	*Balaenoptera physalus*
	XP_010849037.1	*Bison bison bison*
	XP_019835758.1	*Bos indicus*
	XP_014333273.1	*Bos mutus*
	XP_002694753.1	*Bos taurus*
	XP_044788051.2	*Bubalus bubalis*
	XP_055408047.1	*Bubalus carabanensis*
	XP_052512485.1	*Budorcas taxicolor*
	XP_045360937.1	*Camelus bactrianus*
	XP_031313937.1	*Camelus dromedarius*
	XP_032342629.1	*Camelus ferus*
	XP_017917192.1	*Capra hircus*
	XP_008065929.1	*Carlito syrichta*
	XP_020035251.1	*Castor canadensis*
	XP_013010449.2	*Cavia porcellus*
	XP_017386044.1	*Cebus imitator*
	XP_014648168.1	*Ceratotherium simum simum*
	XP_011890147.1	*Cercocebus atys*
	XP_043292624.1	*Cervus canadensis*
	XP_043753983.1	*Cervus elaphus*

	XP_013359316.1	*Chinchilla lanigera*
	XP_037863969.1	*Chlorocebus sabaeus*
	XP_037672086.1	*Choloepus didactylus*
	XP_006860370.1	*Chrysochloris asiatica*
	XP_004690247.1	*Condylura cristata*
	XP_027261832.1	*Cricetulus griseus*
	XP_004464837.1	*Dasypus novemcinctus*
	XP_022439277.2	*Delphinapterus leucas*
	XP_024411697.1	*Desmodus rotundus*
	KAF5922330.1	*Diceros bicornis minor*
	XP_012868142.1	*Dipodomys ordii*
	XP_042556891.1	*Dipodomys spectabilis*
	XP_004704851.2	*Echinops telfairi*
	XP_006878923.1	*Elephantulus edwardii*
	XP_027998602.2	*Eptesicus fuscus*
	XP_014705578.1	*Equus asinus*
	XP_005608486.1	*Equus caballus*
	XP_008532329.1	*Equus przewalskii*
	XP_046536380.1	*Equus quagga*
	XP_007527656.1	*Erinaceus europaeus*
	XP_008570998.1	*Galeopterus variegatus*
	XP_030719681.1	*Globicephala melas*
	XP_030858817.1	*Gorilla gorilla gorilla*
	XP_028624554.1	*Grammomys surdaster*
	XP_021112154.1	*Heterocephalus glaber*
	XP_019501947.1	*Hipposideros armiger*
	NP_689862.1	*Homo sapiens*
	XP_032013789.1	*Hylobates moloch*
	XP_013220622.1	*Ictidomys tridecemlineatus*
	XP_026975717.1	*Lagenorhynchus obliquidens*
	XP_045389908.1	*Lemur catta*
	XP_007463056.1	*Lipotes vexillifer*
	XP_015298622.1	*Macaca fascicularis*
	XP_014982106.1	*Macaca mulatta*

	XP_011756363.1	*Macaca nemestrina*
	XP_011832183.1	*Mandrillus leucophaeus*
	XP_036849776.1	*Manis javanica*
	XP_036782841.1	*Manis pentadactyla*
	XP_027789017.1	*Marmota flaviventris*
	XP_015351559.1	*Marmota marmota marmota*
	XP_046291041.1	*Marmota monax*
	XP_031197317.1	*Mastomys coucha*
	XP_021488182.1	*Meriones unguiculatus*
	XP_040592723.1	*Mesocricetus auratus*
	XP_012627935.1	*Microcebus murinus*
	XP_050000357.1	*Microtus fortis*
	XP_005345906.1	*Microtus ochrogaster*
	XP_041506420.1	*Microtus oregoni*
	XP_016053249.1	*Miniopterus natalensis*
	XP_036131820.1	*Molossus molossus*
	XP_029061253.1	*Monodon monoceros*
	XP_055269114.1	*Moschus berezovskii*
	KAB0350904.1	*Muntiacus muntjak*
	KAB0383612.1	*Muntiacus reevesi*
	XP_021026554.1	*Mus caroli*
	NP_001297542.1	*Mus musculus*
	XP_021076382.1	*Mus pahari*
	XP_048293193.1	*Myodes glareolus*
	XP_014392232.1	*Myotis brandtii*
	XP_006755827.1	*Myotis davidii*
	XP_014313467.1	*Myotis lucifugus*
	XP_036202388.1	*Myotis myotis*
	XP_008821194.1	*Nannospalax galili*
	XP_024591477.1	*Neophocaena asiaeorientalis asiaeorientalis*
	OBS81377.1	*Neotoma lepida*
	XP_030685115.1	*Nomascus leucogenys*
	XP_053463708.1	*Nycticebus coucang*

	XP_040838305.1	*Ochotona curzoniae*
	XP_004584250.1	*Ochotona princeps*
	XP_020744846.1	*Odocoileus virginianus texanus*
	XP_036044342.1	*Onychomys torridus*
	XP_033290579.2	*Orcinus orca*
	XP_007655108.1	*Ornithorhynchus anatinus*
	XP_040109476.1	*Oryx dammah*
	XP_023369861.1	*Otolemur garnettii*
	XP_042087400.1	*Ovis aries*
	XP_008953137.3	*Pan paniscus*
	XP_009429522.1	*Pan troglodytes*
	XP_009195110.2	*Papio anubis*
	XP_052585135.1	*Peromyscus californicus insignis*
	XP_028746937.1	*Peromyscus leucopus*
	XP_028748613.1	*Peromyscus leucopus*
	XP_006974218.1	*Peromyscus maniculatus bairdii*
	XP_047648512.1	*Phacochoerus africanus*
	XP_032470718.1	*Phocoena sinus*
	XP_051057720.1	*Phodopus roborovskii*
	XP_035868106.1	*Phyllostomus discolor*
	XP_045674728.1	*Phyllostomus hastatus*
	XP_023980672.1	*Physeter catodon*
	XP_023058220.1	*Piliocolobus tephrosceles*
	XP_036296921.1	*Pipistrellus kuhlii*
	XP_054390103.1	*Pongo abelii*
	XP_054310884.1	*Pongo pygmaeus*
	XP_012496968.1	*Propithecus coquereli*
	XP_055477445.1	*Psammomys obesus*
	XP_054434994.1	*Pteronotus parnellii mesoamericanus*
	XP_006908885.1	*Pteropus alecto*
	XP_039737014.1	*Pteropus giganteus*
	XP_023378937.1	*Pteropus vampyrus*
	CAI9162389.1	*Rangifer tarandus platyrhyncus*
	NP_001388006.1	*Rattus norvegicus*

	XP_032743911.1	*Rattus rattus*
	XP_032985081.1	*Rhinolophus ferrumequinum*
	XP_019565962.1	*Rhinolophus sinicus*
	XP_017724198.1	*Rhinopithecus bieti*
	XP_030781188.1	*Rhinopithecus roxellana*
	XP_015999382.1	*Rousettus aegyptiacus*
	XP_010348928.1	*Saimiri boliviensis boliviensis*
	XP_032140324.1	*Sapajus apella*
	XP_047385919.1	*Sciurus carolinensis*
	XP_055002171.1	*Sorex araneus*
	XP_036919525.1	*Sturnira hondurensis*
	XP_049643396.1	*Suncus etruscus*
	XP_003481839.3	*Sus scrofa*
	XP_055153920.1	*Symphalangus syndactylus*
	XP_038610628.1	*Tachyglossus aculeatus*
	XP_025227347.1	*Theropithecus gelada*
	XP_033074897.1	*Trachypithecus francoisi*
	XP_023597697.1	*Trichechus manatus latirostris*
	XP_006152702.1	*Tupaia chinensis*
	XP_019797052.1	*Tursiops truncatus*
	XP_026255032.1	*Urocitellus parryii*
	XP_031536686.1	*Vicugna pacos*

Supplemental Table S2. The number of identified MLKL in each species.

Clade	Species	Number
Invertebrate	*Adineta vaga*	4
	Asterias rubens	4
	Anneissia japonica	2
	Branchiostoma floridae	2
	Patiria miniata	2
	Saccoglossus kowalevskii	2
	Acanthaster planci	1
	Branchiostoma belcheri	1
	Branchiostoma lanceolatum	1
	Ciona intestinalis	1
	Strongylocentrotus purpuratus	1
	Styela clava	1
Cyclostomata	*Petromyzon marinus*	1
Chondrichthyes	*Scyliorhinus canicula*	2
	Scyliorhinus torazame	2
	Stegostoma fasciatum	2
	Amblyraja radiata	1
	Callorhinchus milii	1
	Carcharodon carcharias	1
	Chiloscyllium plagiosum	1
	Chiloscyllium punctatum	1
	Leucoraja erinacea	1
	Pristis pectinata	1
	Rhincodon typus	1
Actinopterygii	*Trematomus bernacchii*	4
	Astatotilapia calliptera	3
	Dissostichus mawsoni	3
	Haplochromis burtoni	3
	Maylandia zebra	3
	Oncorhynchus mykiss	3
	Oreochromis niloticus	3

	Oryzias javanicus	3
	Puntigrus tetrazona	3
	Salmo salar	3
	Salmo trutta	3
	Salvelinus alpinus	3
	Salvelinus namaycush	3
	Simochromis diagramma	3
	Sparus aurata	3
	Thunnus maccoyii	3
	Xiphophorus hellerii	3
	Xiphophorus maculatus	3
	Acipenser ruthenus	2
	Archocentrus centrarchus	2
	Betta splendens	2
	Carassius auratus	2
	Carassius gibelio	2
	Cheilinus undulatus	2
	Collichthys lucidus	2
	Conger conger	2
	Cyprinus carpio	2
	Dissostichus eleginoides	2
	Fundulus heteroclitus	2
	Hippoglossus hippoglossus	2
	Kryptolebias marmoratus	2
	Lates calcarifer	2
	Mugil cephalus	2
	Neolamprologus brichardi	2
	Oncorhynchus gorbuscha	2
	Oncorhynchus keta	2
	Oncorhynchus kisutch	2
	Oncorhynchus nerka	2
	Oncorhynchus tshawytscha	2
	Oreochromis aureus	2
	Oryzias latipes	2

	Oryzias melastigma	2
	Parambassis ranga	2
	Perca flavescens	2
	Perca fluviatilis	2
	Poecilia formosa	2
	Poecilia mexicana	2
	Poecilia reticulata	2
	Pogonophryne albipinna	2
	Polyodon spathula	2
	Salarias fasciatus	2
	Salvelinus fontinalis	2
	Thunnus albacares	2
	Xiphophorus couchianus	2
	Acanthochromis polyacanthus	1
	Acanthopagrus latus	1
	Albula glossodonta	1
	Albula goreensis	1
	Aldrovandia affinis	1
	Alosa sapidissima	1
	Ameiurus melas	1
	Amphiprion ocellaris	1
	Anabarilius grahami	1
	Anabas testudineus	1
	Anarrhichthys ocellatus	1
	Anguilla anguilla	1
	Anoplopoma fimbria	1
	Astyanax mexicanus	1
	Austrofundulus limnaeus	1
	Boleophthalmus pectinirostris	1
	Brienomyrus brachyistius	1
	Chaenocephalus aceratus	1
	Channa argus	1
	Chanos chanos	1
	Chelmon rostratus	1

	Clarias gariepinus	1
	Clarias magur	1
	Clupea harengus	1
	Colossoma macropomum	1
	Coregonus clupeaformis	1
	Coregonus sp. 'balchen'	1
	Cottoperca gobio	1
	Ctenopharyngodon idella	1
	Cyclopterus lumpus	1
	Cynoglossus semilaevis	1
	Cyprinodon variegatus	1
	Denticeps clupeoides	1
	Dicentrarchus labrax	1
	Echeneis naucrates	1
	Electrophorus electricus	1
	Epinephelus fuscoguttatus	1
	Epinephelus lanceolatus	1
	Epinephelus moara	1
	Erpetoichthys calabaricus	1
	Esox lucius	1
	Etheostoma spectabile	1
	Gadus morhua	1
	Gambusia affinis	1
	Gasterosteus aculeatus aculeatus	1
	Girardinichthys multiradiatus	1
	Gymnodraco acuticeps	1
	Hemibagrus wyckioides	1
	Hippoglossus stenolepis	1
	Hypomesus transpacificus	1
	Ictalurus furcatus	1
	Ictalurus punctatus	1
	Labeo rohita	1
	Labrus bergylta	1
	Larimichthys crocea	1

	Lepisosteus oculatus	1
	Liparis tanakae	1
	Mastacembelus armatus	1
	Megalobrama amblycephala	1
	Megalops cyprinoides	1
	Melanotaenia boesemani	1
	Menidia menidia	1
	Micropterus dolomieu	1
	Micropterus salmoides	1
	Misgurnus anguillicaudatus	1
	Monopterus albus	1
	Morone saxatilis	1
	Muraenolepis orangiensis	1
	Myripristis murdjan	1
	Myxocyprinus asiaticus	1
	Nematolebias whitei	1
	Nothobranchius furzeri	1
	Notolabrus celidotus	1
	Notothenia coriiceps	1
	Onychostoma macrolepis	1
	Pangasianodon gigas	1
	Pangasianodon hypophthalmus	1
	Pangasius djambal	1
	Paralichthys olivaceus	1
	Paramormyrops kingsleyae	1
	Periophthalmus magnuspinnatus	1
	Pimephales promelas	1
	Plectropomus leopardus	1
	Pleuronectes platessa	1
	Poecilia latipinna	1
	Poeciliopsis prolifica	1
	Polypterus senegalus	1
	Prochilodus magdalenae	1
	Pseudochaenichthys georgianus	1

	Pundamilia nyererei	1
	Pungitius pungitius	1
	Pygocentrus nattereri	1
	Sander lucioperca	1
	Scatophagus argus	1
	Scleropages formosus	1
	Scomber japonicus	1
	Scophthalmus maximus	1
	Scortum barcoo	1
	Sebastes umbrosus	1
	Seriola dumerili	1
	Seriola lalandi dorsalis	1
	Silurus asotus	1
	Silurus meridionalis	1
	Siniperca chuatsi	1
	Sinocyclocheilus anshuiensis	1
	Sinocyclocheilus grahami	1
	Sinocyclocheilus rhinocerous	1
	Solea senegalensis	1
	Sphaeramia orbicularis	1
	Stegastes partitus	1
	Synaphobranchus kaupii	1
	Synchiropus splendidus	1
	Tachysurus fulvidraco	1
	Tetraodon nigroviridis	1
	Thalassophryne amazonica	1
	Toxotes jaculatrix	1
	Triplophysa rosa	1
	Triplophysa tibetana	1
	Xiphias gladius	1
	Xyrauchen texanus	1
Coelacanthimorpha	*Latimeria chalumnae*	1
Dipnomorpha	*Protopterus annectens*	2
Amphibia	*Rana temporaria*	3

	Bombina bombina	2
	Bufo bufo	2
	Engystomops pustulosus	2
	Spea bombifrons	2
	Xenopus laevis	2
	Bufo gargarizans	1
	Eleutherodactylus coqui	1
	Hymenochirus boettgeri	1
	Microcaecilia unicolor	1
	Nanorana parkeri	1
	Pleurodeles waltl	1
	Rhinatrema bivittatum	1
	Xenopus tropicalis	1
Squamata	*Gekko japonicus*	2
	Zootoca vivipara	2
	Anolis carolinensis	1
	Crotalus tigris	1
	Eublepharis macularius	1
	Hemicordylus capensis	1
	Lacerta agilis	1
	Lerista edwardsae	1
	Naja naja	1
	Notechis scutatus	1
	Pantherophis guttatus	1
	Phrynocephalus forsythii	1
	Podarcis muralis	1
	Podarcis raffonei	1
	Pogona vitticeps	1
	Protobothrops mucrosquamatus	1
	Pseudonaja textilis	1
	Python bivittatus	1
	Sphaerodactylus townsendi	1
	Thamnophis elegans	1
	Varanus komodoensis	1

Testudines	*Gopherus evgoodei*	2
	Malaclemys terrapin pileata	2
	Pelodiscus sinensis	2
	Caretta caretta	1
	Chelonia mydas	1
	Chelydra serpentina	1
	Chrysemys picta bellii	1
	Dermochelys coriacea	1
	Gopherus flavomarginatus	1
	Mauremys mutica	1
	Mauremys reevesii	1
	Platysternon megacephalum	1
	Trachemys scripta elegans	1
Crocodilia	*Crocodylus porosus*	2
Aves	*Acanthisitta chloris*	1
	Accipiter gentilis	1
	Acrocephalus arundinaceus	1
	Aegithalos caudatus	1
	Aegotheles bennettii	1
	Agelaius phoeniceus	1
	Aix galericulata	1
	Alaudala cheleensis	1
	Alca torda	1
	Aleadryas rufinucha	1
	Alectura lathami	1
	Alopecoenas beccarii	1
	Amazona guildingii	1
	Anas platyrhynchos	1
	Anhinga anhinga	1
	Anhinga rufa	1
	Anser cygnoides	1
	Anseranas semipalmata	1
	Anthoscopus minutus	1
	Antrostomus carolinensis	1

	Apaloderma vittatum	1
	Aphelocoma coerulescens	1
	Aptenodytes forsteri	1
	Aptenodytes patagonicus	1
	Apteryx mantelli mantelli	1
	Apteryx rowi	1
	Apus apus	1
	Aquila chrysaetos chrysaetos	1
	Aramus guarauna	1
	Ardeotis kori	1
	Arenaria interpres	1
	Asarcornis scutulata	1
	Athene cunicularia	1
	Atlantisia rogersi	1
	Atrichornis clamosus	1
	Aythya fuligula	1
	Balaeniceps rex	1
	Balearica regulorum gibbericeps	1
	Bambusicola thoracicus	1
	Baryphthengus martii	1
	Bombycilla garrulus	1
	Brachypodius atriceps	1
	Brachypteracias leptosomus	1
	Bucco capensis	1
	Buceros rhinoceros silvestris	1
	Bucorvus abyssinicus	1
	Buphagus erythrorhynchus	1
	Burhinus bistriatus	1
	Calcarius ornatus	1
	Calidris pugnax	1
	Callaeas wilsoni	1
	Caloenas nicobarica	1
	Calonectris borealis	1
	Calypte anna	1

	Calyptomena viridis	1
	Camarhynchus parvulus	1
	Campylorhamphus procurvoides	1
	Cardinalis cardinalis	1
	Cariama cristata	1
	Casuarius casuarius	1
	Cathartes aura	1
	Catharus fuscescens	1
	Catharus ustulatus	1
	Centrocercus urophasianus	1
	Centropus bengalensis	1
	Centropus unirufus	1
	Cephalopterus ornatus	1
	Cepphus grylle	1
	Cercotrichas coryphoeus	1
	Certhia brachydactyla	1
	Certhia familiaris	1
	Cettia cetti	1
	Ceuthmochares aereus	1
	Chaetops frenatus	1
	Chaetorhynchus papuensis	1
	Chaetura pelagica	1
	Charadrius vociferus	1
	Chauna torquata	1
	Chionis minor	1
	Chiroxiphia lanceolata	1
	Chlamydotis macqueenii	1
	Chloebia gouldiae	1
	Chloroceryle aenea	1
	Chloropsis cyanopogon	1
	Chordeiles acutipennis	1
	Chroicocephalus maculipennis	1
	Chunga burmeisteri	1
	Ciccaba nigrolineata	1

	Ciconia maguari	1
	Cinclus mexicanus	1
	Circaetus pectoralis	1
	Cisticola juncidis	1
	Climacteris rufus	1
	Cnemophilus loriae	1
	Cochlearius cochlearius	1
	Colinus virginianus	1
	Colius striatus	1
	Columba livia	1
	Copsychus sechellarum	1
	Corapipo altera	1
	Corvus brachyrhynchos	1
	Corvus moneduloides	1
	Corythaeola cristata	1
	Corythaixoides concolor	1
	Coturnix japonica	1
	Crotophaga sulcirostris	1
	Cuculus canorus	1
	Cyanistes caeruleus	1
	Cygnus atratus	1
	Cygnus olor	1
	Daphoenositta chrysoptera	1
	Dasyornis broadbenti	1
	Dicaeum eximium	1
	Dicrurus megarhynchus	1
	Donacobius atricapilla	1
	Dromaius novaehollandiae	1
	Dromas ardeola	1
	Drymodes brunneopygia	1
	Dryobates pubescens	1
	Dryoscopus gambensis	1
	Edolisoma coerulescens	1
	Elachura formosa	1

	Emberiza fucata	1
	Empidonax traillii	1
	Erithacus rubecula	1
	Erpornis zantholeuca	1
	Eubucco bourcierii	1
	Eudyptes chrysocome	1
	Eudyptes moseleyi	1
	Eudyptes pachyrhynchus	1
	Eudyptes robustus	1
	Eudyptes schlegeli	1
	Eudyptes sclateri	1
	Eudyptula minor	1
	Eudyptula novaehollandiae	1
	Eulacestoma nigropectus	1
	Eurypyga helias	1
	Eurystomus gularis	1
	Falco cherrug	1
	Falco naumanni	1
	Falco peregrinus	1
	Falcunculus frontatus	1
	Ficedula albicollis	1
	Formicarius rufipectus	1
	Fregata magnificens	1
	Fregetta grallaria	1
	Fulmarus glacialis	1
	Furnarius figulus	1
	Galbula dea	1
	Gallus gallus	1
	Gavia stellata	1
	Geococcyx californianus	1
	Geospiza fortis	1
	Glareola pratincola	1
	Glaucidium brasilianum	1
	Grallaria varia	1

	Grantiella picta	1
	Grus americana	1
	Gymnogyps californianus	1
	Gymnorhina tibicen	1
	Halcyon senegalensis	1
	Haliaeetus albicilla	1
	Harpia harpyja	1
	Heliornis fulica	1
	Hemiprocne comata	1
	Herpetotheres cachinnans	1
	Himantopus himantopus	1
	Hippolais icterina	1
	Hirundo rustica	1
	Horornis vulcanius	1
	Hylia prasina	1
	Hypocryptadius cinnamomeus	1
	Ibidorhyncha struthersii	1
	Ifrita kowaldi	1
	Illadopsis cleaveri	1
	Indicator indicator	1
	Indicator maculatus	1
	Irena cyanogastra	1
	Jacana jacana	1
	Lagopus leucura	1
	Lagopus muta	1
	Lamprotornis superbus	1
	Lanius ludovicianus	1
	Leiothrix lutea	1
	Lepidothrix coronata	1
	Leptocoma aspasia	1
	Leptosomus discolor	1
	Leucopsar rothschildi	1
	Limosa lapponica baueri	1
	Locustella ochotensis	1

	Lonchura striata domestica	1
	Lophotis ruficrista	1
	Loxia curvirostra	1
	Loxia leucoptera	1
	Machaerirhynchus nigripectus	1
	Malurus elegans	1
	Manacus candei	1
	Manacus vitellinus	1
	Megadyptes antipodes antipodes	1
	Melanocharis versteri	1
	Meleagris gallopavo	1
	Melopsittacus undulatus	1
	Melospiza melodia maxima	1
	Melozone crissalis	1
	Menura novaehollandiae	1
	Merops nubicus	1
	Mesembrinibis cayennensis	1
	Mesitornis unicolor	1
	Mionectes macconnelli	1
	Mohoua ochrocephala	1
	Molothrus ater	1
	Motacilla alba	1
	Motacilla alba alba	1
	Myiagra hebetior	1
	Myiozetetes cayanensis	1
	Mystacornis crossleyi	1
	Neodrepanis coruscans	1
	Neopelma chrysocephalum	1
	Neopipo cinnamomea	1
	Nesospiza acunhae	1
	Nestor notabilis	1
	Nicator chloris	1
	Nipponia nippon	1
	Nothocercus julius	1

	Nothocercus nigrocapillus	1
	Nothoprocta ornata	1
	Nothoprocta pentlandii	1
	Nothoprocta perdicaria	1
	Notiomystis cincta	1
	Numida meleagris	1
	Nyctibius bracteatus	1
	Nyctibius grandis	1
	Nycticryphes semicollaris	1
	Nyctiprogne leucopyga	1
	Oceanites oceanicus	1
	Oceanodroma tethys	1
	Odontophorus gujanensis	1
	Oenanthe oenanthe	1
	Onychorhynchus coronatus	1
	Onychostruthus taczanowskii	1
	Opisthocomus hoazin	1
	Oreocharis arfaki	1
	Oreotrochilus melanogaster	1
	Origma solitaria	1
	Oriolus oriolus	1
	Orthonyx spaldingii	1
	Oxylabes madagascariensis	1
	Oxyruncus cristatus	1
	Oxyura jamaicensis	1
	Pachycephala philippinensis	1
	Pachyramphus minor	1
	Pandion haliaetus	1
	Panurus biarmicus	1
	Pardalotus punctatus	1
	Parus major	1
	Passer montanus	1
	Passerina amoena	1
	Patagioenas fasciata monilis	1

	Pedionomus torquatus	1
	Pelecanoides urinatrix	1
	Pelecanus crispus	1
	Penelope pileata	1
	Peucedramus taeniatus	1
	Phaethon lepturus	1
	Phaetusa simplex	1
	Phainopepla nitens	1
	Phalacrocorax carbo	1
	Phasianus colchicus	1
	Pheucticus melanocephalus	1
	Phoenicopterus ruber ruber	1
	Piaya cayana	1
	Picathartes gymnocephalus	1
	Pipra filicauda	1
	Piprites chloris	1
	Pitangus sulphuratus	1
	Pitta sordida	1
	Platysteira castanea	1
	Ploceus nigricollis	1
	Pluvianellus socialis	1
	Podargus strigoides	1
	Podiceps cristatus	1
	Podilymbus podiceps	1
	Poecile atricapillus	1
	Polioptila caerulea	1
	Pomatorhinus ruficollis	1
	Pomatostomus ruficeps	1
	Probosciger aterrimus	1
	Promerops cafer	1
	Prunella fulvescens	1
	Prunella himalayana	1
	Pseudopodoces humilis	1
	Psilopogon haemacephalus	1

	Psophia crepitans	1
	Pterocles burchelli	1
	Pterocles gutturalis	1
	Pteruthius melanotis	1
	Ptilonorhynchus violaceus	1
	Pycnonotus jocosus	1
	Pygoscelis adeliae	1
	Pygoscelis antarcticus	1
	Pygoscelis papua	1
	Pyrgilauda ruficollis	1
	Quiscalus mexicanus	1
	Ramphastos sulfuratus	1
	Regulus satrapa	1
	Rhabdornis inornatus	1
	Rhadina sibilatrix	1
	Rhagologus leucostigma	1
	Rhinopomastus cyanomelas	1
	Rhinoptilus africanus	1
	Rhipidura dahli	1
	Rhodinocichla rosea	1
	Rhynochetos jubatus	1
	Rissa tridactyla	1
	Rostratula benghalensis	1
	Rynchops niger	1
	Sagittarius serpentarius	1
	Sakesphorus luctuosus	1
	Sapayoa aenigma	1
	Sclerurus mexicanus	1
	Scopus umbretta	1
	Scytalopus superciliaris	1
	Semnornis frantzii	1
	Serilophus lunatus	1
	Serinus canaria	1
	Setophaga kirtlandii	1

	Sinosuthora webbiana	1
	Sitta europaea	1
	Smithornis capensis	1
	Spheniscus magellanicus	1
	Spheniscus mendiculus	1
	Spizaetus tyrannus	1
	Steatornis caripensis	1
	Stercorarius parasiticus	1
	Sterrhoptilus dennistouni	1
	Strigops habroptila	1
	Struthidea cinerea	1
	Struthio camelus australis	1
	Sturnus vulgaris	1
	Sula dactylatra	1
	Sylvia atricapilla	1
	Sylvia borin	1
	Sylvietta virens	1
	Syrrhaptes paradoxus	1
	Tachuris rubrigastra	1
	Taeniopygia guttata	1
	Tauraco erythrolophus	1
	Thalassarche chlororhynchos	1
	Thinocorus orbignyianus	1
	Thryothorus ludovicianus	1
	Tichodroma muraria	1
	Tinamus guttatus	1
	Todus mexicanus	1
	Toxostoma redivivum	1
	Tricholaema leucomelas	1
	Trogon melanurus	1
	Turnix velox	1
	Tympanuchus pallidicinctus	1
	Tyrannus savana	1
	Tyto alba	1

	Upupa epops	1
	Uria aalge	1
	Urocolius indicus	1
	Urocynchramus pylzowi	1
	Vidua chalybeata	1
	Vidua macroura	1
	Vireo altiloquus	1
	Zapornia atra	1
	Zonotrichia albicollis	1
	Zosterops hypoxanthus	1
Mammalia	*Peromyscus leucopus*	2
	Acomys russatus	1
	Aotus nancymaae	1
	Apodemus sylvaticus	1
	Artibeus jamaicensis	1
	Arvicanthis niloticus	1
	Arvicola amphibius	1
	Balaenoptera physalus	1
	Bison bison bison	1
	Bos indicus	1
	Bos mutus	1
	Bos taurus	1
	Bubalus bubalis	1
	Bubalus carabanensis	1
	Budorcas taxicolor	1
	Camelus bactrianus	1
	Camelus dromedarius	1
	Camelus ferus	1
	Capra hircus	1
	Carlito syrichta	1
	Castor canadensis	1
	Cavia porcellus	1
	Cebus imitator	1
	Ceratotherium simum simum	1

	Cercocebus atys	1
	Cervus canadensis	1
	Cervus elaphus	1
	Chinchilla lanigera	1
	Chlorocebus sabaeus	1
	Choloepus didactylus	1
	Chrysochloris asiatica	1
	Condylura cristata	1
	Cricetulus griseus	1
	Dasypus novemcinctus	1
	Delphinapterus leucas	1
	Desmodus rotundus	1
	Diceros bicornis minor	1
	Dipodomys ordii	1
	Dipodomys spectabilis	1
	Echinops telfairi	1
	Elephantulus edwardii	1
	Eptesicus fuscus	1
	Equus asinus	1
	Equus caballus	1
	Equus przewalskii	1
	Equus quagga	1
	Erinaceus europaeus	1
	Galeopterus variegatus	1
	Globicephala melas	1
	Gorilla gorilla gorilla	1
	Grammomys surdaster	1
	Heterocephalus glaber	1
	Hipposideros armiger	1
	Homo sapiens	1
	Hylobates moloch	1
	Ictidomys tridecemlineatus	1
	Lagenorhynchus obliquidens	1
	Lemur catta	1

	Lipotes vexillifer	1
	Macaca fascicularis	1
	Macaca mulatta	1
	Macaca nemestrina	1
	Mandrillus leucophaeus	1
	Manis javanica	1
	Manis pentadactyla	1
	Marmota flaviventris	1
	Marmota marmota marmota	1
	Marmota monax	1
	Mastomys coucha	1
	Meriones unguiculatus	1
	Mesocricetus auratus	1
	Microcebus murinus	1
	Microtus fortis	1
	Microtus ochrogaster	1
	Microtus oregoni	1
	Miniopterus natalensis	1
	Molossus molossus	1
	Monodon monoceros	1
	Moschus berezovskii	1
	Muntiacus muntjak	1
	Muntiacus reevesi	1
	Mus caroli	1
	Mus musculus	1
	Mus pahari	1
	Myodes glareolus	1
	Myotis brandtii	1
	Myotis davidii	1
	Myotis lucifugus	1
	Myotis myotis	1
	Nannospalax galili	1
	Neophocaena asiaeorientalis asiaeorientalis	1

	Neotoma lepida	1
	Nomascus leucogenys	1
	Nycticebus coucang	1
	Ochotona curzoniae	1
	Ochotona princeps	1
	Odocoileus virginianus texanus	1
	Onychomys torridus	1
	Orcinus orca	1
	Ornithorhynchus anatinus	1
	Oryx dammah	1
	Otolemur garnettii	1
	Ovis aries	1
	Pan paniscus	1
	Pan troglodytes	1
	Papio anubis	1
	Peromyscus californicus insignis	1
	Peromyscus maniculatus bairdii	1
	Phacochoerus africanus	1
	Phocoena sinus	1
	Phodopus roborovskii	1
	Phyllostomus discolor	1
	Phyllostomus hastatus	1
	Physeter catodon	1
	Piliocolobus tephrosceles	1
	Pipistrellus kuhlii	1
	Pongo abelii	1
	Pongo pygmaeus	1
	Propithecus coquereli	1
	Psammomys obesus	1
	Pteronotus parnellii mesoamericanus	1
	Pteropus alecto	1
	Pteropus giganteus	1
	Pteropus vampyrus	1
	Rangifer tarandus platyrhyncus	1

	Rattus norvegicus	1
	Rattus rattus	1
	Rhinolophus ferrumequinum	1
	Rhinolophus sinicus	1
	Rhinopithecus bieti	1
	Rhinopithecus roxellana	1
	Rousettus aegyptiacus	1
	Saimiri boliviensis boliviensis	1
	Sapajus apella	1
	Sciurus carolinensis	1
	Sorex araneus	1
	Sturnira hondurensis	1
	Suncus etruscus	1
	Sus scrofa	1
	Symphalangus syndactylus	1
	Tachyglossus aculeatus	1
	Theropithecus gelada	1
	Trachypithecus francoisi	1
	Trichechus manatus latirostris	1
	Tupaia chinensis	1
	Tursiops truncatus	1
	Urocitellus parryii	1
	Vicugna pacos	1

Supplemental Table S3. The primers used for mutagenesis.

Primer	Sequence (5’-3’)
AvMLKL-1-NTD-I10A-forward	5’-TATCAGTCATACATAGCGCTTATGATGTGG-3’
AvMLKL-1-NTD-I10A-reverse	5’-AGCGCTATGTATGACTGATATTGGATCCAT-3’
AvMLKL-1-NTD-I17A-forward	5’-GATGTGGGAATGGCAGCTCGAAAAAGC-3’
AvMLKL-1-NTD-I17A-reverse	5’-GCTGCCATTCCCACATCATAGATGCTA-3’
AvMLKL-1-NTD-E22A-forward	5’-TTCGAAAAAGCGTGGCACACGTAAAA-3’
AvMLKL-1-NTD-E22A-reverse	5’-GCCACGCTTTTTCGAATTGCCATTCC-3’
AvMLKL-1-NTD-C31S-forward	5’-AAAGCTAATGAAAAACAATCTATAAGACTTG-3’
AvMLKL-1-NTD-C31S-reverse	5’-GATTGTTTTTCATTAGCTTTTACGTGTTCC-3’
AvMLKL-1-NTD-R37A-forward	5’-ATAAGACTTGCAGAAGCAATCGATGCG-3’
AvMLKL-1-NTD-R37A-reverse	5’-GCTTCTGCAAGTCTTATACATTGTTTT-3’
AvMLKL-1-NTD-TK77-78AA-forward	5’-TGCTTGGAATTTGTTGCAGCATTTACCGAT-3’
AvMLKL-1-NTD-TK77-78AA-reverse	5’-GCTGCAACAAATTCCAAGCATTGTTCGATA-3’
AvMLKL-1-NTD-F99A-forward	5’-CAAAATTTCAAACAACATGCTGAAGAACTA-3’
AvMLKL-1-NTD-F99A-reverse	5’-GCATGTTGTTTGAAATTTTGATTGCGAAAA-3’
AvMLKL-1-NTD-N103A-forward	5’-CAACATTTTGAAGAACTAGCTCTTCAACTG-3’
AvMLKL-1-NTD-N103A-reverse	5’-GCTAGTTCTTCAAAATGTTGTTTGAAATTT-3’
AvMLKL-1-NTD-L106A-forward	5’-GAAGAACTAAATCTTCAAGCGTCACAAAGC-3’
AvMLKL-1-NTD-L106A-reverse	5’-GCTTGAAGATTTAGTTCTTCAAAATGTTGT-3’
AvMLKL-1-NTD-L113A-forward	5’-CAAAGCGCTGTTGATGCTAATTTAGGT-3’
AvMLKL-1-NTD-L113A-reverse	5’-GCATCAACAGCGCTTTGTGACAGTTGA-3’
AvMLKL-1-NTD-L115A-forward	5’-CGCTGTTGATCTTAATGCAGGTATCAAT-3’
AvMLKL-1-NTD-L115A-reverse	5’-GCATTAAGATCAACAGCGCTTTGTGACA-3’
AvMLKL-1-NTD-pCAGGS-FLAG-C-forward	5’-CATCATTTTGGCAAAGAATTCATGGATCCAATATCAGTCATACATAGCA-3’
AvMLKL-1-NTD-pCAGGS-FLAG-C-reverse	5’-GTAATCCATAGATCTGCTAGCATTCAATCTCGATTGAATAGTATTCA-3’
CiMLKL-NTD-pCAGGS-FLAG-C-forward	5’-CATCATTTTGGCAAAGAATTCATGGCTTTGGAAATTGTTGCGTGT-3’
CiMLKL-NTD-pCAGGS-FLAG-C-reverse	5’-GTAATCCATAGATCTGCTAGCATTAAGCATATGTTGTATGTCTTTCA-3’
BfMLKL-2-NTD-pCAGGS-FLAG-C-forward	5’-CATCATTTTGGCAAAGAATTCATGATTTGGTGGCGTGAACTTCCG-3’
BfMLKL-2-NTD-pCAGGS-FLAG-C-reverse	5’-GTAATCCATAGATCTGCTAGCTTCCTTAAACATGGCGTCAACTGTCCC-3’
BlMLKL NTD-pCAGGS-FLAG-C-forward	5’-CATCATTTTGGCAAAGAATTCATGTCTGACCCTATATCTGTTCTATCTACC-3’

BlMLKL NTD-pCAGGS-FLAG-C-reverse	5'-GTAATCCATAGATCTGCTAGCTCTCGTCTCTTCGTTAAACATGGCGT-3'
ApMLKL NTD-pCAGGS-FLAG-C-forward	5'-CATCATTTTGGCAAAGAATTCATGGAGGTTGTTAAACTGGCCGTAG-3'
ApMLKL NTD-pCAGGS-FLAG-C-reverse	5'-GTAATCCATAGATCTGCTAGCGTCATTCTTGAATTGATACATAATCTC-3'
PmMLKL-1-NTD-pCAGGS-FLAG-C-forward	5'-CATCATTTTGGCAAAGAATTCATGGACGTCTTTTCACTGGCACAG-3'
PmMLKL-1-NTD-pCAGGS-FLAG-C-reverse	5'-GTAATCCATAGATCTGCTAGCAATGAGGTCACGTTCATCCTCCTC-3'
